# Constitutive Turbodomains enhance expansion and antitumor activity of allogeneic BCMA CAR T cells in preclinical models

**DOI:** 10.1126/sciadv.adg8694

**Published:** 2023-08-04

**Authors:** Regina J. Lin, Janette Sutton, Trevor Bentley, Diego A. Vargas-Inchaustegui, Duy Nguyen, Hsin-Yuan Cheng, Hayung Yoon, Thomas J. Van Blarcom, Barbra J. Sasu, Siler H. Panowski, Cesar Sommer

**Affiliations:** Allogene Therapeutics Inc., 210 E. Grand Avenue, South San Francisco, CA 94080, USA.

## Abstract

The magnitude of CAR T cell expansion has been associated with clinical efficacy. Although cytokines can augment CAR T cell proliferation, systemically administered cytokines can result in toxicities. To gain the benefits of cytokine signaling while mitigating toxicities, we designed constitutively active synthetic cytokine receptor chimeras (constitutive Turbodomains) that signal in a CAR T cell–specific manner. The modular design of Turbodomains enables diverse cytokine signaling outputs from a single homodimeric receptor chimera and allows multiplexing of different cytokine signals. Turbodomains containing an IL-2/15Rβ–derived signaling domain closely mimicked IL-15 signaling and enhanced CAR T cell potency. Allogeneic TurboCAR T cells targeting BCMA showed no evidence of aberrant proliferation yet displayed enhanced expansion and antitumor activity, prolonging survival and preventing extramedullary relapses in mouse models. These results illustrate the potential of constitutive Turbodomains to achieve selective potentiation of CAR T cells and demonstrate the safety and efficacy of allogeneic BCMA TurboCAR T cells, supporting clinical evaluation in multiple myeloma.

## Teaser

Synthetic cytokine receptor chimeras providing IL-15 signaling potentiate allogeneic CAR T cells in preclinical models.

## INTRODUCTION

Chimeric antigen receptor (CAR) T cell therapies have revolutionized cancer treatment, with six CAR T cell products for aggressive hematologic malignancies receiving regulatory approval by the U.S. Food and Drug Administration so far. With the exception of B cell acute lymphoblastic leukemia, however, a substantial proportion of patients do not respond to treatment or relapse within the first year (*1*, *2*). The mechanisms of resistance remain incompletely elucidated and may include target antigen loss or downmodulation, suppression of T cell effector function, and low CAR T cell fitness (*3*). In vivo expansion of CAR T cells has been identified as a major determinant of clinical outcomes (*4*–*6*), prompting attempts to devise strategies to boost the survival and potency of the infused therapeutic cells.

Accumulating evidence suggests that cytokine signaling may improve CAR T cell survival and antitumor function. Increased serum cytokine levels, most notably interleukin-15 (IL-15), are associated with CAR T cell expansion and disease remission (*7*). Rapid elevation of IL-15 in the tumor microenvironment, associated with T cell infiltration and activity, was recently found in patients responding favorably to axicabtagene ciloleucel (axi-cel) (*8*). In preclinical cancer models, exogenous cytokine supplementation or transgenic overexpression of IL-15 was shown to augment the proliferative capacity and antitumor activity of CAR T cells (*9*–*11*).

While high-dose cytokine administration could enhance CAR T cell efficacy, particularly in patients with insufficient lymphodepletion, its benefits may be limited by potentially severe toxicities and pleiotropic effects on bystander host immune cells (*12*). In addition to safety concerns, systemic cytokine administration or the extracellular release of cytokines from the infused cells may promote early rejection of allogeneic CAR T cells (*13*), hindering any potential advantages to off-the-shelf cellular therapies. Restricting cytokine signaling to allogeneic CAR T cells may therefore increase their engraftment and effector function, potentially leading to improved tumor control, while simultaneously circumventing possible side effects of free cytokines.

We recently reported the design of inducible chimeric cytokine receptors that mimic the signaling of the native receptors and whose activation can be controlled by clinically safe pharmacological agents (*14*). Leveraging the modular nature of this platform, here, we describe the development of ligand-independent, constitutively active chimeric cytokine receptors (constitutive Turbodomains). We show that constitutive Turbodomains can potentiate CAR T cell function and evaluate the preclinical activity of allogeneic B cell maturation antigen (BCMA) CAR T cells engineered to express a Turbodomain mimicking IL-15 signaling for the treatment of multiple myeloma.

## RESULTS

### Turbodomains are homodimeric cytokine receptor chimeras that provide strong, constitutive, and diverse signaling

**Fig. 1.**
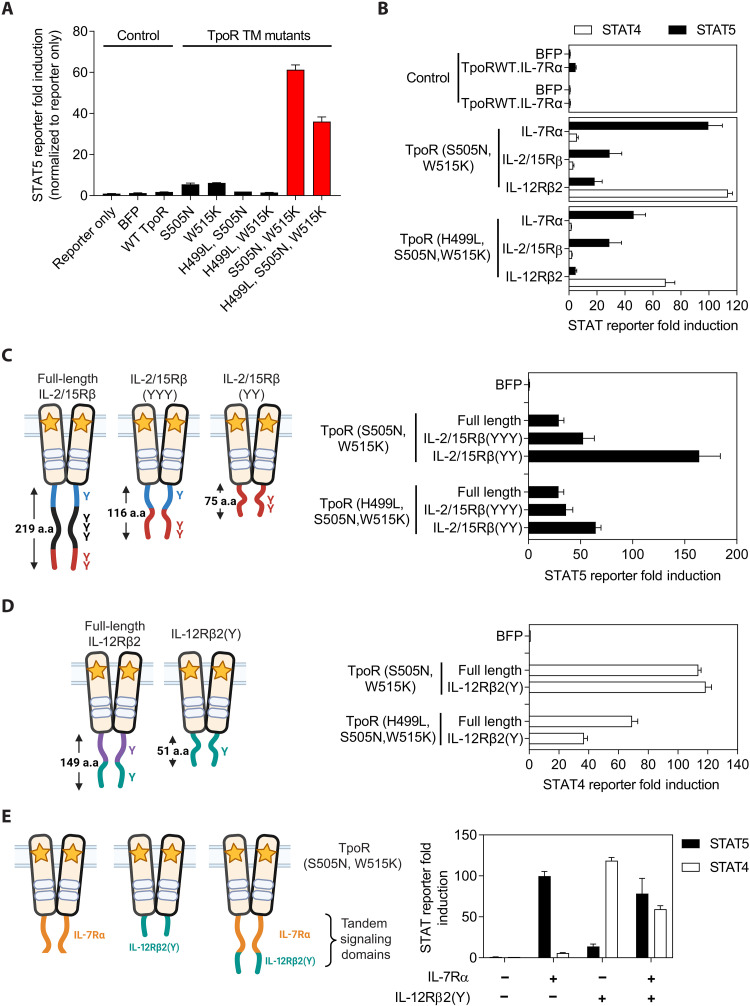
Turbodomains are designed to provide strong, constitutive, and diverse cytokine signaling with a compact cargo size. (**A**) Using a HEK293T luciferase reporter assay and a Turbodomain containing an IL-7Rα signaling domain, TpoR TM domain mutants were screened to identify those capable of enforcing strong, constitutive receptor dimerization and signaling. Mutant variants that generated the strongest cytokine signal, as measured by STAT5-driven luciferase reporter activity, are highlighted by the red bars. (**B**) Turbodomains bearing full-length signaling domains derived from the indicated cytokine receptors preferentially induced downstream STAT-driven luciferase reporter activity as expected of the parental cytokine receptor. (**C** and **D**) Truncations of the full-length IL-2/15Rβ (C) or IL-12Rβ2 (D) signaling domains were generated by retaining only the indicated tyrosine residues, and signaling activity was assessed in a HEK293T reporter assay. (**E**) Signaling domains derived from distinct cytokine receptors can be fused in tandem to simultaneously activate multiple STAT pathways from a single homodimeric Turbodomain. In all experiments, firefly luciferase activity was first normalized to Renilla luciferase activity, and then STAT response element fold induction was calculated by normalizing to untransfected and untreated controls. Bar graphs represent means ± SEM of triplicates from one representative experiment of three. WT, wild type; a.a., amino acid.

The inducible Turbo (iTurbo) platform (*14*) was used as the basis for designing constitutively active cytokine receptor chimeras (constitutive Turbodomains). Specifically, a Turbodomain composed of a thrombopoietin receptor (TpoR)–derived transmembrane (TM) domain and Janus kinase (JAK) binding domain and an IL-7Rα–derived signaling domain (*14*) was selected for additional engineering. We first introduced point mutations to the TpoR TM domain that had been reported to induce ligand-independent receptor dimerization and activation (fig. S1) (*15*–*17*). A signal transducer and activator of transcription 5 (STAT5) reporter assay was used to identify mutations that could induce constitutive activity from the IL-7Rα signaling domain. Compared to a Turbodomain with wild-type TpoR TM domain, Turbodomains containing either the S505N or W515K point mutation induced a minimal increase in STAT5 reporter activity, but the combination of both point mutations synergistically improved signal strength (Fig. 1A). The combination of the H499L point mutation with either the single or double S505N, W515K point mutations dampened signal strength. The S505N, W515K and the H499L, S505N, W515K mutants generated the strongest STAT5 signal, and these constitutive Turbodomains were selected for further evaluation and engineering.

To explore alternative signaling outputs of Turbodomains, the IL-2/15Rβ and IL-12Rβ2 intracellular domains were also tested. Fusion of TpoR (S505N, W515K) and TpoR (H499L, S505N, W515K) to the IL-7Rα or IL-2/15Rβ signaling domains resulted in preferential activation of STAT5, whereas fusion to the IL-12Rβ2 signaling domain resulted in preferential activation of STAT4 (Fig. 1B), demonstrating that constitutive Turbodomains are capable of flexible signaling outputs and can mimic signaling of the parental cytokine receptors. As full-length signaling domains can contribute substantially to vector cargo and contain negative regulatory motifs that can reduce signaling activity (*14*, *18*), the signaling domains were modified to optimize the size and signal strength of Turbodomains. Compared to the full-length IL-2/15Rβ signaling domain that contains six tyrosine residues capable of mediating downstream signaling, similar signal strength was achieved with the truncated IL-2/15Rβ(YYY) variant, and removal of the negative regulatory membrane-proximal tyrosine residue, involved in IL-2R endocytosis (*19*, *20*) [IL-2/15Rβ(YY) variant], further enhanced signaling (Fig. 1C). Following a similar strategy, a minimal signaling domain for IL-12Rβ2 was engineered. The smaller IL-12Rβ2(Y) signaling domain activated STAT4 efficiently, with the potential to recapitulate the signal strength of full-length IL-12Rβ2 (Fig. 1D). A similar optimization of IL-7Rα was not performed because earlier studies had identified a minimal signaling domain that resulted in only a modest reduction in cargo size with no enhancement in signal strength (*14*).

To evaluate whether signaling from multiple cytokine receptors can be recapitulated by a single homodimeric Turbodomain, signaling domains from two different cytokine receptors were fused in tandem. While Turbodomains containing either the IL-7Rα or minimal IL-12Rβ2(Y) signaling domain preferentially activated STAT5 or STAT4, respectively, Turbodomains containing the tandem fusion activated both STAT pathways simultaneously (Fig. 1E).

### TurboCAR T cells exhibit enhanced cytotoxicity, expansion, and polyfunctionality

**Fig. 2.**
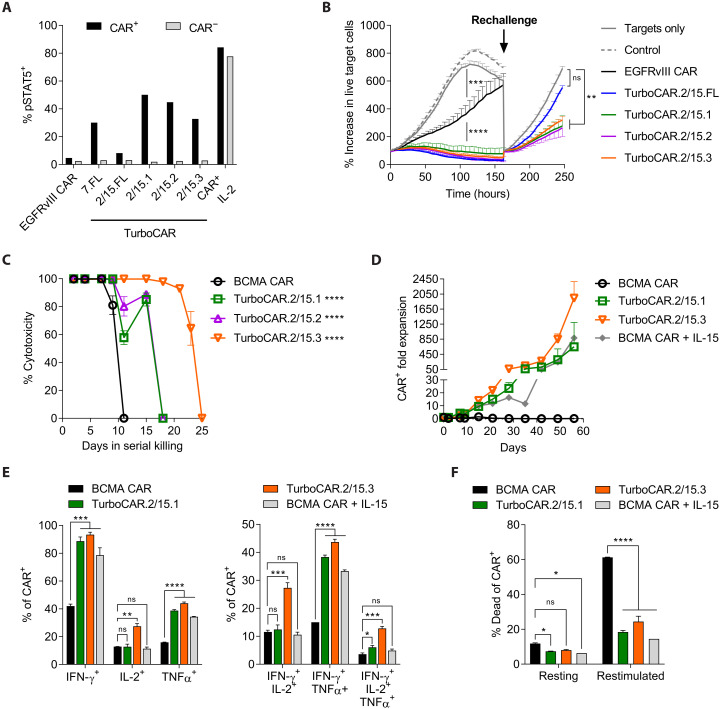
TurboCAR T cells show increased expansion and sustained cytolytic activity. (**A**) STAT5 activation as seen by percentage of pSTAT5-expressing cells was detected in the CAR^+^ but not the CAR^−^ fraction of T cells transduced with the TurboCAR constructs. By contrast, CAR T cells that had been treated with exogenous IL-2 showed STAT5 activation in both the CAR^+^ and CAR^−^ fractions. EGFRvIII CAR^−^ T cells were used as background gating controls. Data are shown for one representative of three donors. (**B**) Cytotoxicity of TurboCAR T cells expressing the Turbo.2/15 variants against nuclear GFP-labeled U87KO-EGFRvIII target cells was assessed in an Incucyte assay at an effector–to–target (E:T) ratio of 1:3. Where indicated, CAR T cells were rechallenged with fresh target cells. The percentage increase in live target cells was determined by normalizing target cell counts at each time point to that at time = 0. Data are means ± SEM of triplicate wells from one representative of two donors. (**C**) Long-term cytotoxicity of BCMA TurboCAR T cells was assessed using MM.1S-Luc-GFP target cells at an E:T of 10:1. Fresh target cells were added every 2 to 3 days, and target cell killing was determined by luminescence readout. (**D**) Target cell–driven expansion of BCMA TurboCAR T cells serially challenged with MM.1S-Luc-GFP target cells. Data are presented as means ± SEM of triplicates from one of two donors. (**E** and **F**) On day 7, TurboCAR T cells showed improved effector cytokine expression and greater polyfunctionality by intracellular cytokine staining (E) as well as reduced activation-induced cell death (F). Data are presented as means ± SD of duplicates from one of two donors. Statistical significance determined by ordinary one-way analysis of variance (ANOVA) with Dunnett’s test [(B), (C), and (E)] or two-way ANOVA with Dunnett’s test (F). *P < 0.05, **P < 0.01, ***P < 0.001, and ****P < 0.0001; ns, not significant. IFN-γ, interferon-γ; TNFα, tumor necrosis factor–α.

To determine whether constitutive Turbodomains can improve the activity of CAR T cells, TurboCAR T cells were generated using bicistronic lentiviral vectors designed for the coexpression of a selected Turbodomain and a second generation CAR directed toward epidermal growth factor receptor variant III (EGFRvIII) (*21*) or BCMA (*11*) (fig. S2A). Compared to the EGFRvIII CAR construct, most TurboCAR lentiviral constructs showed lower initial transduction efficiency, but transduced cells enriched and expanded to similar CAR^+^ T cell yields, with the exception of TurboCAR T cells expressing the Turbo.2/15.FL variant (fig. S2, B to D). Increased STAT5 activation was detected selectively in the CAR^+^ cell population but not in the CAR^−^ cell population, demonstrating that Turbodomain signaling is specific to cells coexpressing the CAR and Turbodomain (Fig. 2A and fig. S2E). An in vitro cytotoxicity assay with EGFRvIII TurboCAR T cells was conducted using the Incucyte Live Cell Imaging System to assess the relative potency of different Turbodomains. EGFRvIII TurboCAR T cells with the Turbo.7.FL variant (fig. S3A) or with any of the Turbo.2/15 variants tested (Fig. 2B) showed improved initial cytotoxicity compared to EGFRvIII CAR T cells and continued to inhibit target cell growth following rechallenge. By contrast, EGFRvIII TurboCAR T cells expressing the Turbo.12 variants exhibited no functional enhancement and were therefore deprioritized (fig. S3B).

The activity of Turbo.7.FL and Turbo.2/15 variants was further characterized in the context of a BCMA CAR. As multiple myeloma target cells are nonadherent, they could not be accurately imaged using the IncuCyte system. Therefore, an alternate and more stringent long-term killing assay was performed in which TurboCAR T cells were rechallenged with fresh target cells every 2 or 3 days. While BCMA TurboCAR T cells with the Turbo.7.FL variant did not display improvements in activity in this assay (fig. S4), expression of Turbo.2/15 variants prolonged cytotoxicity relative to BCMA CAR T cells (Fig. 2C), with the Turbo.2/15.3 variant displaying the highest potency. In response to serial target exposure, BCMA TurboCAR T cells with Turbo.2/15.3 mediated robust target-induced expansion comparable to BCMA CAR T cells supplemented with exogenous IL-15 (Fig. 2D). On a per-cell level, BCMA TurboCAR T cells expressing this Turbodomain variant also exhibited higher expression of effector cytokines and greater polyfunctionality (Fig. 2E) and displayed decreased activation-induced cell death (Fig. 2F) compared to BCMA CAR T cells.

### IL-2/15R–derived Turbodomains induce low mTORC1 activity and more closely mimic IL-15 than IL-2 signaling

**Fig. 3.**
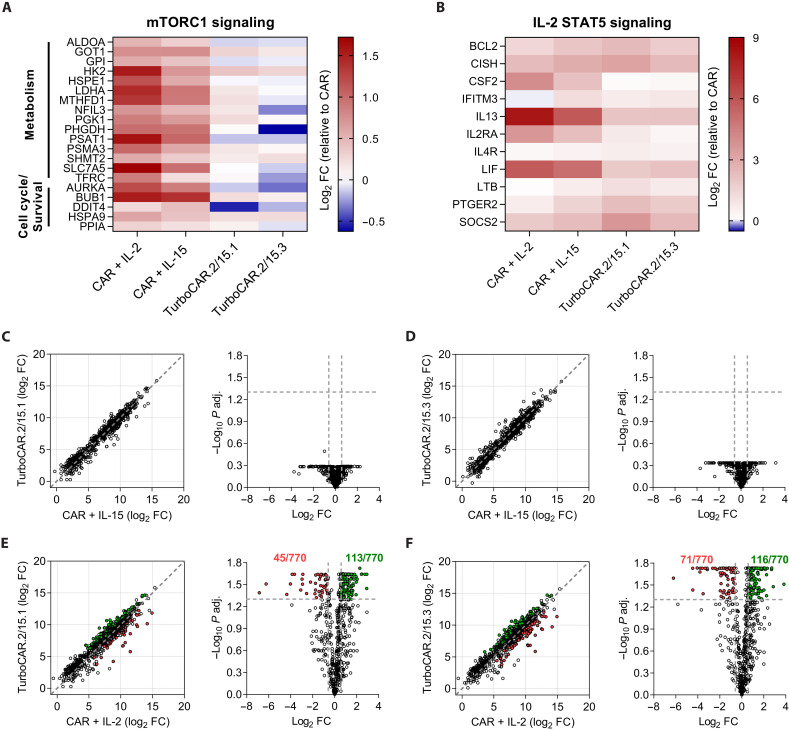
IL-2/15R–derived Turbodomains more closely mimic IL-15 than IL-2 signaling. Gene expression analysis was performed using the NanoString nCounter Human CAR T Characterization panel. Heatmap of genes up-regulated by cytokine treatment or Turbodomain signaling associated with mTORC1 signaling (**A**) and STAT5 signaling (**B**) relative to cytokine untreated CAR T cells. Scatter plots (left) and volcano plots (right) comparing gene expression profiles of IL-15–treated CAR T cells with TurboCAR T cells expressing either Turbo.2/15.1 (**C**) or Turbo.2/15.3 (**D**). Scatter plots (left) and volcano plots (right) comparing gene expression profiles of IL-2–treated CAR T cells with TurboCAR T cells expressing either Turbo.2/15.1 (**E**) or Turbo.2/15.3 (**F**). Genes significantly up-regulated and down-regulated by TurboCAR T cells are shown in green and red, respectively. FC, fold change.

Because IL-2 and IL-15 both use IL-2Rβ and the common gamma chain (γ_c_), TurboCAR T cells expressing Turbo.2/15 variants were compared to CAR T cells cultured in the presence of these cytokines to determine similarities and differences to both. As TurboCAR T cells were routinely produced and expanded in IL-2, initial expansion was carried out in IL-2 only or in a combination of IL-2 and IL-15. At the end of expansion, CAR^+^ cells were fluorescence-activated cell sorting (FACS)–sorted and cultured with IL-2, IL-15, or in the absence of cytokines for an additional 2 days to further enrich for Turbodomain- or cytokine-induced gene signatures before NanoString gene expression profiling (fig. S5A). The gene expression data of TurboCAR T cells and cytokine-treated CAR T cells were first normalized to CAR T cells that were cultured in the absence of cytokines for the final 2 days. Meta-analysis was then performed to identify gene clusters and pathways that were similarly or differentially represented as a result of cytokine treatment or Turbodomain signaling compared to cytokine-untreated CAR T cells (fig. S5B). Of the six gene clusters identified (fig. S5C), cytokine treatment and Turbodomain signaling resulted in up-regulation of genes associated with mammalian target of rapamycin complex (mTORC) signaling (Fig. 3A) and IL-2 STAT5 signaling (Fig. 3B), further corroborating that Turbo.2/15 variants can productively and constitutively activate the STAT5 pathway, albeit less strongly than exogenous cytokines (fig. S2E). Genes in the mTORC pathway (associated with metabolism, cell cycle, and survival) were more strongly up-regulated in response to cytokine treatment compared to Turbodomain signaling, with IL-2 being more potent than IL-15 (Fig. 3A). Differential gene expression analysis between TurboCAR T cells and cytokine-treated CAR T cells was then conducted to evaluate whether expression of the Turbo.2/15 variants more closely recapitulated IL-2 or IL-15 signaling. Among 770 genes evaluated, a comparison of TurboCAR T cells to IL-15–treated CAR T cells revealed highly similar gene expression profiles, with no significantly differentially expressed genes (Fig. 3, C and D). By contrast, compared to IL-2–treated CAR T cells, TurboCAR T cells differentially expressed 158 and 187 genes, respectively (Fig. 3, E and F). Gene set enrichment analysis identified these differentially expressed genes to be associated with mTORC1 signaling and/or glycolysis, with IL-2–treated CAR T cells being more metabolically active and TurboCAR T cells remaining more quiescent (fig. S5D). Together, these data suggest that signaling from Turbo.2/15 variants is different than IL-2 and closely mimics IL-15.

### TurboCAR T cells do not kill or proliferate in the absence of target and exogenous cytokine stimulation

**Fig. 4.**
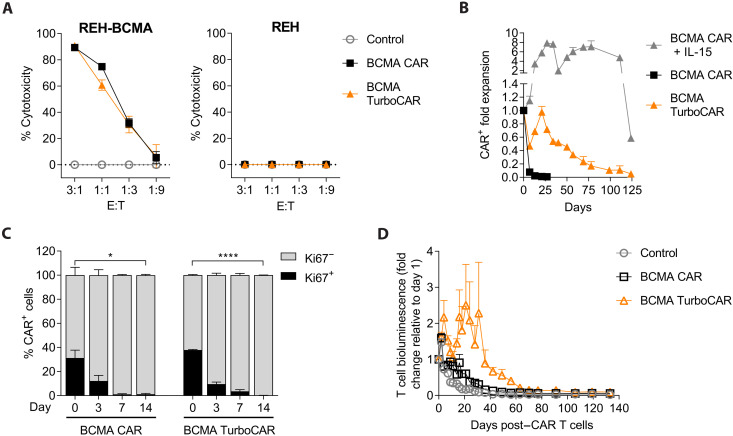
Allogeneic BCMA TurboCAR T cells do not expand in the absence of target and exogenous cytokines. (**A**) Specific cytotoxic activity of allogeneic BCMA TurboCAR T cells compared to BCMA CAR T cells and nontransduced cells was assessed after coculture with luciferase-labeled REH cells with or without overexpression of BCMA at decreasing E:T ratios and measuring the residual luciferase activity at 24 hours. Graphs represent percentage of cell lysis relative to target cells cultured alone (means ± SEM of triplicates from one representative of two donors). (**B**) Proliferative capacity of BCMA TurboCAR T cells cultured in the absence of cytokines or target cells was assessed by counting total viable cells over time. Fold expansion at each time point was calculated relative to day 0 and is shown as means + SEM of duplicate wells for each CAR T cell product. (**C**) Frequency of BCMA TurboCAR T cells expressing the proliferation marker Ki67 was estimated during the first 14 days of the cytokine and target-independent proliferation assay using flow cytometry. Data are shown as means + SEM of three donors. Statistical significance determined by a Student’s *t* test (*P < 0.05 and ****P < 0.0001). (**D**) CBR-labeled BCMA TurboCAR T cells were infused in nontumor–bearing NSG mice, and persistence was monitored by measuring whole-body bioluminescence for several months. BCMA CAR T cells and nontransduced (control) T cells that had been similarly labeled were also tested for comparison. Results are expressed as fold change from the bioluminescence measured on the day of T cell administration (means + SEM, *n* = 7 to 10).

Turbodomains are designed to promote CAR T cell function and survival following activation. IL-15 signaling leading to autonomous CAR T cell proliferation in the absence of target stimulation, if present, could pose unacceptable safety risks to patients and compromise potential therapeutic benefits. To thoroughly characterize the effector function and safety profile of TurboCAR T cells in the context of an allogeneic therapeutic product, we generated BCMA TurboCAR T cells with targeted disruption of the T cell receptor alpha constant (*TRAC*) and *CD52 *genes using healthy donor–derived peripheral blood mononuclear cells (PBMCs), as described previously (*11*). For these studies, we chose to use BCMA TurboCAR T cells expressing the Turbo.2/15.3 variant given the superior activity seen in vitro (Fig. 2). Robust cell expansion (fig. S6A), effective transduction (fig. S6B), and efficient knockout of *TRAC* and *CD52 *genes (fig. S6C) were seen across the three donors tested. Compared to BCMA CAR T cells, BCMA TurboCAR T cells had a higher frequency of long-lived stem cell memory (T_SCM_) cells (fig. S6D). Similar to BCMA CAR T cells, target cell killing and cytokine secretion by BCMA TurboCAR T cells were dependent on the presence of antigen (Fig. 4A and fig. S6E). To assess the risk of uncontrolled proliferation, BCMA TurboCAR T cells were cultured in assay medium without addition of exogenous cytokines or target cells. BCMA TurboCAR T cells showed negligible expansion during the first weeks and gradually declined over time (Fig. 4B and fig. S7). While cell numbers declined slowly, examination of the cell proliferation marker Ki67 revealed a rapid and substantial decrease from 30 to 40% in post-thaw CAR^+^ cells to undetectable levels by day 14 of culture (Fig. 4C). Cell expansion and persistence in the absence of target and cytokine stimulation were also assessed in vivo following administration of click beetle red luciferase (CBR)–labeled BCMA TurboCAR T cells to nontumor-bearing mice. Whole-body bioluminescence imaging allowed an indirect measurement of CAR T cell levels that was not restricted to circulating CAR T cells and could measure persistence or expansion in tissues. No sign of aberrant BCMA TurboCAR T cell expansion was observed (Fig. 4D). Cells persisted for approximately 30 days and then gradually declined, reaching 1/10 of the signal measured following infusion by day 100 compared to BCMA CAR T cells reaching this level at approximately day 50 and nontransduced control cells by day 30. The signal continued to gradually diminish for the duration of the study, and no signs of toxicity were observed in any of the mice. Overall, these results indicate that allogeneic BCMA TurboCAR T cells do not expand in the absence of stimulation by exogenous cytokines or target cells and do not kill in a target-independent manner.

### Long-term persistence of allogeneic BCMA TurboCAR T cells requires regular antigen stimulation and can be controlled by safety switches

**Fig. 5.**
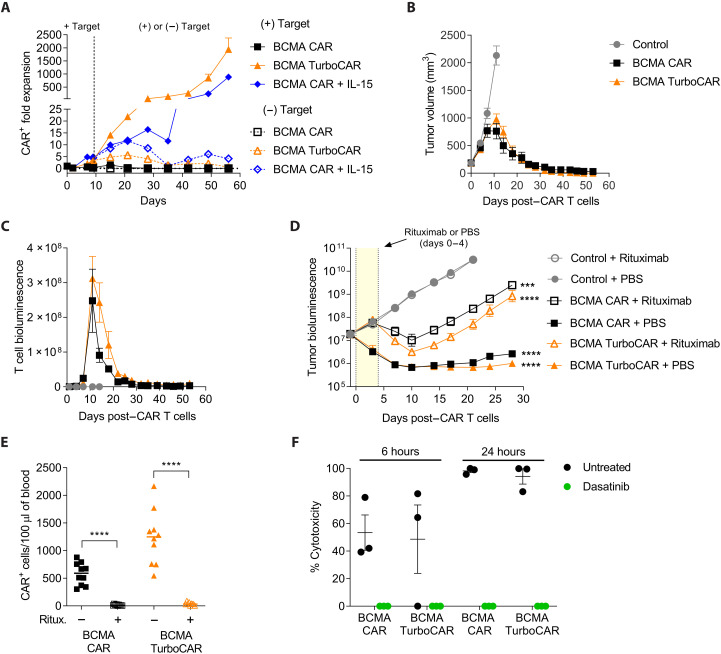
Long-term survival and activity of allogeneic BCMA TurboCAR T cells are target dependent and can be controlled by safety switches. (**A**) Proliferation of BCMA TurboCAR T cells was measured under continuous stimulation with target cells [(+) Target] and after withdrawal of the target [(−) Target] on day 9 following initial stimulation using flow cytometry. Data are means + SEM of duplicates from one representative of two donors. (**B** and **C**) Target-induced expansion and persistence of BCMA TurboCAR T cells were evaluated in NSG mice bearing subcutaneous tumors. (B) Molp-8 tumor cells were injected on the right flank and tumor volume was monitored using calipers. (C) Mice received 0.5 × 10^6^ CBR^+^CAR^+^ cells mixed with 9.5 × 10^6^ unlabeled CAR^+^ cells, and persistence was evaluated by bioluminescence. Data are means ± SEM (*n* = 7 to 8). (**D**) NSG mice engrafted with 5 × 10^6^ luciferase-labeled MM.1S cells were treated with a single dose of 3 × 10^6^ CAR^+^ cells followed by rituximab (five daily doses of 10 mg/kg, i.p.) or vehicle control. Tumor burden was monitored by bioluminescence and data are shown as means ± SEM (*n* = 9 to 10). Statistics were performed using repeated measures (RM) one-way ANOVA with Dunnett’s test (***P < 0.001 and ****P < 0.0001). A paired *t* test was performed for individual CAR T cell–treated groups with and without rituximab. (**E**) Circulating CAR^+^ cells were quantitated by flow cytometry 3 days after the last dose of rituximab. Statistics were performed using a paired *t* test (****P < 0.0001). (**F**) Inhibition of cytotoxicity by dasatinib was determined by first activating BCMA TurboCAR T cells in the presence of luciferase-labeled MM.1S target cells and then culturing the activated cells with fresh target cells in the presence or absence of dasatinib (100 nM). Graphs represent percentage of cell lysis after 6 and 24 hours. Data are means ± SEM (*n* = 3 donors).

To investigate if the proliferative advantage conferred by Turbodomain signaling requires parallel and periodic CAR engagement, BCMA TurboCAR T cells were exposed to target cells for 9 days and were then transferred to wells that received either fresh target cells or T cell assay medium alone, and this step was repeated for several days. In response to antigen restimulation, BCMA TurboCAR T cells continued to expand (Fig. 5A). Comparable expansion was observed for BCMA CAR T cells in the presence but not in the absence of exogenous IL-15 (Fig. 5A). Following removal of target cells on day 9, BCMA TurboCAR T cells ceased to expand and underwent a gradual decline. We next investigated the persistence of luciferase-labeled BCMA TurboCAR T cells in mice bearing subcutaneous BCMA^+^ tumors. Complete tumor control and sustained antitumor activity through day 53 was attained by both the BCMA CAR T and BCMA TurboCAR T cells (Fig. 5B). The start of tumor regression on day 11 coincided with peak CAR T cell expansion, after which CAR T cells showed a progressive decline (Fig. 5C). In this model, where tumor eradication was achieved, both BCMA CAR T and TurboCAR T cells showed comparable expansion and contraction kinetics that were controlled by the abundance of target cells. As expected for target-dependent activity and persistence, BCMA TurboCAR T cells were progressively localized to the tumor site (fig. S8). Collectively, these data indicate that, similar to BCMA CAR T cells, BCMA TurboCAR T cells expand and exhibit antitumor activity with subsequent contraction after elimination of target cells.

Additional control of CAR T cell activity in the event of unacceptable toxicities can be achieved with the inclusion of a suicide gene (*22*, *23*). The CAR expressed by BCMA TurboCAR T cells was engineered to contain a compact “off-switch” between the hinge and the scFv that can be activated by the anti-CD20 antibody rituximab, enabling selective elimination of the infused cells (*24*). In vitro, BCMA TurboCAR T cells were effectively depleted in the presence of rituximab and effector natural killer cells by antibody-dependent cellular cytotoxicity in a dose-dependent manner and to the same extent as BCMA CAR T cells (fig. S9A). Similarly, BCMA TurboCAR T cells showed sensitivity to rituximab in a complement-dependent cytotoxicity assay (fig. S9B). The ability of rituximab to abrogate the antitumor effect of BCMA TurboCAR T cells was also examined in an orthotopic mouse model of multiple myeloma. NOD scid gamma (NSG) mice engrafted with luciferase-labeled MM.1S cells were randomly assigned to treatment groups that received an effective dose of 3 × 10^6^ CAR T or TurboCAR T cells and were then administered either rituximab or vehicle control. Tumor growth was observed in mice receiving nontransduced control T cells coadministered either rituximab or vehicle control. Infusion of BCMA CAR T cells or BCMA TurboCAR T cells significantly reduced tumor burden when coadministered with vehicle but the antitumor response was blunted with administration of rituximab (Fig. 5D). Flow cytometry analysis of peripheral blood revealed a substantial reduction in circulating CAR^+^ cells in the mice administered rituximab relative to vehicle control groups (Fig. 5E). Together, these data indicate that the built-in rituximab off-switch can effectively mediate the depletion of BCMA TurboCAR T cells in vitro and in vivo.

We also evaluated the ability of the tyrosine kinase inhibitor dasatinib to suppress TurboCAR T cell activity, as reported previously (*25*). Whereas BCMA TurboCAR T cells cocultured with target cells and vehicle exhibited 50% and more than 90% target cell lysis within 6 and 24 hours, respectively, this cytotoxic activity was completely suppressed by dasatinib (Fig. 5F). Overall, inhibition of cytolytic activity by dasatinib appeared similar between BCMA TurboCAR T cells and BCMA CAR T cells.

### Allogeneic BCMA TurboCAR T cells exhibit enhanced expansion and antitumor activity

**Fig. 6.**
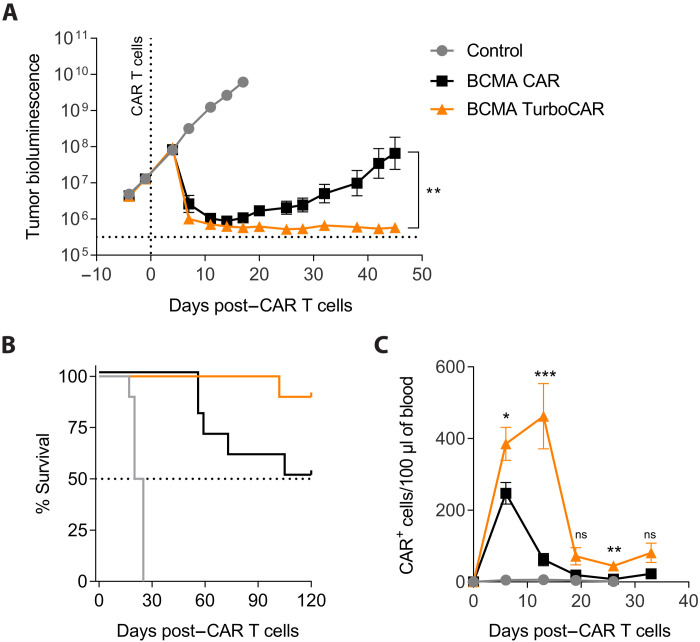
Allogeneic BCMA TurboCAR T cells display increased expansion and long-term antitumor activity in vivo. (**A**) NSG mice received 5 × 10^6^ luciferase-labeled MM.1S cells via tail vein injection, and tumor growth was monitored by bioluminescence (*n* = 10). BCMA TurboCAR T cells (1 × 10^6^) were infused intravenously 15 days later. A two-tailed, paired *t* test for all time points was used for comparison of the different treatments. Data are means ± SEM. (**B**) Survival of mice treated with BCMA TurboCAR T cells, BCMA CAR T cells, or control nontransduced T cells. (**C**) CAR^+^ cells in peripheral blood were enumerated using flow cytometry twice a week. A Student’s *t* test for each time point, without multiple comparison correction, was used for comparison. Data are means ± SEM. *P < 0.05, **P < 0.01, and ***P < 0.001.

We next tested the potency of BCMA TurboCAR T cells in an orthotopic model of multiple myeloma prone to extramedullary relapse. NSG mice engrafted with luciferase-labeled MM.1S cells were randomly assigned to treatment groups and received different doses of TurboCAR T cells. At a limiting dose of 1 × 10^6^ CAR^+^ cells, BCMA TurboCAR T cells but not BCMA CAR T cells were capable of fully controlling tumor growth (Fig. 6A). Lack of tumor control in the BCMA CAR T cell group, as expected, resulted in decreased survival (Fig. 6B). BCMA TurboCAR T cells reached peak levels in peripheral blood around day 13 after treatment (Fig. 6C), which correlated well with maximal decrease in tumor bioluminescence. At higher doses, BCMA CAR T cells showed improved tumor growth control (fig. S10), but mice still showed an increased tendency to relapse with worse survival than their BCMA TurboCAR T cell comparators. Consistent with the in vitro findings, BCMA TurboCAR T cells displayed increased ability to proliferate in vivo compared to BCMA CAR T cells (Fig. 6C and fig. S10). Also, consistent with our in vitro findings (Fig. 2C and fig. S4), BCMA TurboCAR T cells expressing the Turbo.7.FL variant did not achieve enhanced tumor control in vivo, whereas expression of Turbo.2/15.2 and Turbo.2/15.3 improved the antitumor activity, with Turbo.2/15.3 mediating the most durable response (fig. S11A). Compared to mice treated with BCMA CAR T cells, which had relapsed with extramedullary tumors at the end of the study, 9 of 10 mice that had received BCMA TurboCAR T cells expressing Turbo.2/15.3 remained tumor free (fig. S11B). The activity of Turbo.2/15.1 was assessed alongside the most active Turbo.2/15.3 variant in a separate study. Again, BCMA TurboCAR T cells expressing Turbo.2/15.3 provided superior tumor control (fig. S11C), which correlated with enhanced CAR T cell expansion and persistence in the peripheral blood (fig. S11D). Together, Turbo.2/15 variants improved the antitumor efficacy in vivo, with Turbo.2/15.3 mediating the most potent and durable response. Collectively, our findings demonstrate that allogeneic BCMA TurboCAR T cells derived from healthy donor T cells have significantly enhanced potency in preclinical models of multiple myeloma and hold great therapeutic potential.

## DISCUSSION

Early CAR T cell expansion following adoptive cell transfer has been recognized as a critical determinant of clinical efficacy and remission durability in patients with hematologic malignancies. Improving the replicative capacity of CAR T cells could therefore increase the likelihood and durability of responses. We demonstrate that engineering CAR T cells with synthetic Turbodomains providing constitutive cytokine signaling is an effective strategy to enhance target-induced cell expansion and antitumor activity. Our findings suggest that Turbodomains augment CAR T cell responses most effectively when signals 1 and 2 are concurrently delivered by the CAR following antigen recognition. Turbodomains do not lead to unrestricted proliferation or toxicities commonly associated with systemic administration of cytokines. Even after robust activation, sustained expansion and long-term survival of BCMA TurboCAR T cells are still dependent on periodic antigen stimulation. The strict dependence on antigen for functionality and prolonged persistence and the sensitivity of the cells to off-switches that rely on commonly used drugs support a suitable efficacy and safety profile for BCMA TurboCAR T cells.

T cell–potentiating cytokines, such as IL-2, IL-15, IL-7, and IL-12, signal through heterodimeric native receptors. Constitutive Turbodomains not only recapitulate the signaling of native cytokine receptors but also provide additional flexibility for a wide range of tunable, user-programmable, and multiplexable signaling outputs. Furthermore, Turbodomains offer the advantage of a compact homodimeric design, allowing them to be feasibly integrated into CAR expression vectors. Although a larger cargo from the addition of a Turbodomain lowers initial transduction efficiency, constitutive CAR T cell–specific Turbodomain signaling can promote preferential expansion and enrichment, such that CAR percentage and yield in the TurboCAR T cell product are comparable to those of conventional CAR T cells. Among the Turbodomains examined, expression of Turbo.2/15.FL resulted in poor TurboCAR T cell enrichment and yield, likely because it only weakly activated STAT5 in primary CAR T cells and was also the largest of the constructs. Optimization of Turbo.2/15.FL by removing negative regulatory tyrosine residues from its signaling domain improved STAT5 activation, TurboCAR T cell enrichment, and manufacturability.

IL-2 and IL-15 both use IL-2Rβ and the common γ_c_ to trigger near-indistinguishable downstream signaling pathways (*26*). Yet, IL-2 and IL-15 differentially regulate T cell metabolism and consequently dictate distinct functional outcomes, with IL-2 promoting glycolysis and exhaustion, and IL-15 favoring oxidative phosphorylation and long-term persistence (*27*–*30*). In vitro cytotoxicity assays identified two highly active Turbodomain variants, Turbo.2/15.1 and Turbo.2/15.3, bearing truncated forms of the IL-2/15Rβ signaling domain. In agreement with their lower STAT5 protein phosphorylation levels, gene expression analysis demonstrated that signaling elicited by Turbo.2/15 variants was weaker compared to treatment with exogenous IL-2 or IL-15. Despite differences in signal strengths, Turbo.2/15 variants were found to more closely recapitulate IL-15 rather than IL-2 signaling on a transcriptional level. Furthermore, TurboCAR T cells differed metabolically from IL-2–treated CAR T cells, which strongly activate mTORC1 and glycolysis. In an earlier report comparing IL-2– and IL-15–expanded CAR T cells, reduced mTORC1 signaling and glycolysis were identified as two metabolic features that favor T_SCM_ enrichment and associate with improved survival, persistence, and antitumor activity of IL-15–expanded CAR T cells (*30*). Phenocopying these attributes, BCMA TurboCAR T cells expressing Turbo.2/15 variants demonstrated enhanced target-driven expansion, resistance to activation-induced cell death, increased polyfunctionality, and delayed differentiation. Together, these quantitative and qualitative improvements render TurboCAR T cells superior to conventional CAR T cells.

A previous study evaluated a membrane-bound form of chimeric IL-15 (mbIL15) mimicking the transpresentation of the endogenous cytokine and found sustained CAR T cell persistence independent of CAR signaling (*10*). Prolonged engraftment of mbIL15-CAR T cells in immunodeficient mice was observed, even in the absence of antigen, suggesting that T cell receptor (TCR)–mediated xenograft–versus–host disease may have contributed to the increased persistence of these cells in vivo (*10*). Similar observations have been reported by others after infusion of cytokine-producing CAR^+^ TCRαβ^+^ T cells in immunodeficient mice (*31*, *32*). Our findings using allogeneic TCR-deficient BCMA TurboCAR T cells indicate that the increased potency and longer persistence of the cells result from the integration of CAR and Turbodomain signals in response to antigen exposure. It is possible that the function of the Turbodomain is influenced by CAR T cell activation, and this warrants further investigation. TurboCAR T cells showed a higher content of long-lived T_SCM_ cells after production and achieved superior antitumor control, becoming progressively localized to the tumor in subcutaneous models. Yet, similar to conventional CAR T cells, TurboCAR T cells ceased to expand and showed a progressive decline following antigen withdrawal in vitro or after tumor clearance in vivo. Despite exhibiting higher peak levels in peripheral blood relative to BCMA CAR T cells, the kinetics of TurboCAR T cell expansion and contraction following infusion into mice with disseminated disease was consistent with an antigen-dependent T cell response. Moreover, although TurboCAR T cells appeared to survive better in the absence of antigen and exogenous cytokines, no apparent autonomous growth or unrestricted proliferation was seen in in vitro assays or in vivo models.

One limitation of constitutive Turbodomain technology is the inability to control engineered cytokine signaling and downstream cellular effects, a feature of inducible systems such as chimeric orthogonal cytokine receptors (*33*, *34*) and our previously described iTurbo domains (*14*). The possibility to limit or completely abrogate chimeric cytokine receptor signaling in CAR T cells following administration to patients, for example, by altering the dose and timing of the inducing agent, may increase the therapeutic index of investigational cell therapies, particularly when on-target and off-target effects are not completely defined. For CAR T cells directed to validated targets including CD19 and BCMA, with anticipated toxicities that can be monitored and managed, constitutive Turbodomain signaling offers the possibility to potentiate CAR T cell function without the need for periodic administration of an inducer.

Allogeneic BCMA CAR T cells were recently evaluated in a phase 1 clinical trial and showed encouraging safety and efficacy (*35*). As with autologous products, clinical responses in patients with adequate lymphodepletion were dose dependent and associated with CAR T cell expansion. We showed that BCMA TurboCAR T cells could be derived from healthy donor T cells using the same methodologies currently used for producing allogeneic BCMA CAR T cells (*11*). The compact size of the synthetic Turbodomain allowed its coexpression with a BCMA CAR from a single lentiviral vector, which is compatible with current manufacturing processes. Following repeated antigen stimulation in vitro and after infusion in tumor-bearing mice, allogeneic BCMA TurboCAR T cells had higher expansion and longer persistence relative to conventional BCMA CAR T cells. Turbodomains may therefore support selective expansion of activated TurboCAR T cells in the context of the immune reconstitution that follows lymphodepleting chemotherapy. The functional improvements associated with Turbodomains may be particularly valuable for off-the-shelf products, which are subject to rejection by the host and may necessitate enhanced conditioning regimens for full therapeutic efficacy (*36*). Together, the results presented here illustrate the feasibility, efficacy, and safety of allogeneic BCMA TurboCAR T cells, supporting clinical investigation in relapsed or refractory multiple myeloma.

## MATERIALS AND METHODS

### Cells and cell culture conditions

Cell lines and primary cells were cultured in a humidified incubator at 37°C and 5% CO_2_. The cell lines human embryonic kidney (HEK) 293T, Jurkat, LN229, MM.1S, and REH were obtained from American Type Culture Collection (ATCC) (Manassas, VA). The LN229-EGFRvIII cell line was derived from LN229 by stably overexpressing full-length human EGFRvIII via lentiviral transduction. Molp-8 cells (ACC-569) were obtained from Deutsche Sammlung von Mikroorganismen und Zellkulturen (DSMZ; Braunschweig, Germany). The U87KO-EGFRvIII cell line was a gift from Cellectis SA (Paris, France). Briefly, U87KO-EGFRvIII was derived from the parental cell line, U87MG (ATCC, Manassas, VA), by first knocking out endogenous wild-type EGFR using transcription activator-like effector nucleases (TALEN) and then stably overexpressing full-length human EGFRvIII via lentiviral transduction. TALEN gene editing is a technology pioneered and controlled by Cellectis. Tumor cell lines were engineered to express either luciferase and green fluorescent protein (GFP) using Luc2AGFP lentivirus (AMSbio, Cambridge, MA) or nuclear GFP by using IncuCyte NucLight Green Lentivirus Reagent (Essen Bioscience, Ann Arbor, MI). PBMCs were sourced from Stanford Blood Center (Palo Alto, CA) or STEMCELL Technologies (Vancouver, BC, Canada), and T cells were isolated using the human Pan T Cell Isolation Kit (Miltenyi Biotec, Auburn, CA).

### HEK293T cell reporter assays

A total of 20,000 HEK293T cells were plated into each well of a poly-l-lysine–coated 96-well flat-bottom plate and allowed to adhere overnight. A constitutive cytokine receptor vector (2.5 ng), a STAT response element vector that drives Firefly luciferase (100 ng; Promega, Madison, WI) and Renilla luciferase control reporter vector (1 ng; Promega, Madison, WI) were mixed in a final volume of 5 μl in Opti-MEM (Gibco, Thermo Fisher Scientific, Waltham, MA) (“DNA mix”). Lipofectamine 2000 (0.3 μl; Invitrogen, Thermo Fisher Scientific, Waltham, MA) in 5 μl of Opti-MEM was incubated at room temperature for 5 min and then added to the DNA mix. The mixture was incubated at room temperature for 20 min, and the total volume of 10 μl was added to each well containing HEK293T cells. Twenty-four hours after transfection, cells were left untreated, treated with AP1903 (1 μg/ml; APExBio, Houston, TX), or with the indicated cytokines diluted in serum-free media. At the indicated time points after treatment, reporter activity was determined using the Dual-Glo Luciferase Assay System (Promega, Madison, WI) as per the manufacturer’s instructions. Firefly luciferase activity was first normalized to Renilla luciferase activity, and then STATresponse element fold induction was calculated by normalizing to untransfected controls.

### Construction of lentiviral vectors

Turbodomains were generated by genetic fusion of a short extracellular stub, a TM and JAK2-activating domain, and an intracellular signaling domain. The short extracellular stub, TM, and JAK2-activating domains used were derived from TpoR(478–582) [National Center for Biotechnology Information (NCBI) reference sequence: NP_005364.1] and signaling domains were derived from IL-7Rα(316–459) (NCBI reference sequence: NP_002176.2), IL-2Rβ(333–551) (NCBI reference sequence: NP_001333152.1), and IL-12Rβ2(714–862) (NCBI reference sequence: NP_001361188.1) and are as listed in table S1. CARs directed toward EGFRvIII (*21*) and BCMA (*11*) were generated by fusing the respective scFvs to the CD8α hinge/TM domain fused with 4-1BB and CD3ζ intracellular signaling elements. To facilitate the detection of EGFRvIII CAR–transduced cells, a v5 tag was inserted between the scFv and CD8 hinge. All genes were codon-optimized and synthesized to replace the IRES-Puro cassette in the pLVX-EF1α-IRES-Puro vector (Takara Bio, Mountain View, CA), with Turbodomains cloned upstream of the CAR for coexpression via a porcine teschovirus-1 2A (P2A) self-cleaving peptide. All transfer vectors encoded the transgene(s) under regulatory control of the EF1α promoter and the woodchuck hepatitis virus posttranscriptional regulatory element.

### CAR T cell production

CAR T cells were generated as previously described (*11*). Briefly, T cells were activated immediately after recovery from cryopreservation in X-Vivo 15 medium (Lonza, Basel, Switzerland) supplemented with 10% fetal calf serum (FCS) (GE Healthcare, Pittsburgh, PA) and IL-2 (100 U/ml; Miltenyi Biotec, Auburn, CA) using T cell TransAct (Miltenyi Biotec, Auburn, CA) as recommended by the manufacturer. T cells were transduced with lentiviral supernatant or concentrated lentiviruses 2 days after activation and then cultured in X-Vivo 15 medium supplemented with 5% heat-inactivated human AB serum (Gemini Bio-Products, West Sacramento, CA). For the cell production shown in fig. S6, lentiviral titers for the BCMA CAR and BCMA TurboCAR constructs were 2.1 × 10^9^ and 2.5 × 10^8^ transducing units/ml (TU/ml), respectively. IL-2 was supplemented every 2 or 3 days. Where indicated, at 6 days after activation, T cells were transfected with TRAC (25 μg/ml) and/or CD52 TALEN monomer mRNA (TriLink Biotechnologies, San Diego, CA) using AgilePulse MAX electroporators (BTX, Holliston, MA). TALEN gene editing is a technology pioneered and controlled by Cellectis. At 14 days after activation, depletion of TCRα-positive cells was performed using TCRα/β cell isolation kits (Miltenyi Biotec, Auburn, CA). At 15 days after activation, T cells were cryopreserved in 90% FCS/10% dimethyl sulfoxide using rate-controlled freezing chambers and stored in liquid nitrogen vapor phase. All functional assays were performed with cells after recovery from cryopreservation. CBR-labeled CAR T cells were generated as above, with the exception that T cells were cotransduced with the CAR vector and a CBR-P2A-BFP vector, encoding CBR and blue fluorescent protein (BFP). As controls, T cells were transduced with the CBR-P2A-BFP vector alone. Eight days after initial T cell activation, CBR-transduced CAR T cells and CBR control T cells were enriched by FACS for BFP^+^ CAR^+^ and BFP^+^ cells, respectively, using the BD FACSAria Cell Sorter (BD Biosciences, San Jose, CA). Sorted cells were then expanded further for an additional week before cryopreservation.

### In vitro cytotoxicity assays

For short-term killing assays, 1 × 10^4^ luciferase-expressing target cells (MM.1S cells and REH-BCMA cells) or control (REH cells) were cocultured with CAR^+^ cells at defined ratios in 96-well flat-bottom white-wall plates for 24 hours in 200 μl of assay medium without IL-2. After 24 hours, 100 μl of cell culture supernatant was removed, 100 μl of ONE-Glo reagent was added and luminescence was acquired on the SpectraMax L luminometer. For long-term killing assays, luciferase-expressing MM.1S target cells were added at 1 × 10^4^ per well and cocultured with CAR^+^ cells at a predetermined effector–to–target (E:T) ratio of 10:1 in 96-well white flat-bottom plates for 48 hours in 200 μl of assay medium without IL-2. After 2 days, cells were thoroughly mixed by gentle pipetting and 100 μl of cell suspension was transferred to a new 96-well plate containing 1 × 10^4^ target cells per well. The spent plate was read out by adding 100 μl of ONE-Glo reagent (Promega, Madison, WI), and luminescence was acquired on the SpectraMax L luminometer. This process was repeated 3× per week (i.e., cells were transferred to a new plate containing targets every 2 to 3 days) until cytotoxic activity had ceased. Luminescence background was assessed in wells containing only T cells or media and was found to be negligible. Relative luminescence units (RLUs) were converted to percentage of lysed target cells using the formula 100 × [1 − (RLUtest/RLUcontrol)]. Untreated target cells were used to determine RLU control. Where cytotoxicity was evaluated using the IncuCyte Live Cell Imaging System (Essen Bioscience, Ann Arbor, MI), 5 × 10^3^ nuclear GFP-labeled U87KO-EGFRvIII target cells were seeded and allowed to attach in 96-well black-wall plates with flat clear bottom. CAR^+^ cells were then added at an E:T ratio of 1:3. Cytotoxicity was determined by the change in nucGFP-labeled target cell counts over time.

### Gene expression profiling and analysis

FACS-sorted cells (2 × 10^5^) were spun down, and cell pellets were frozen until RNA was extracted and ran on the nCounter Human CAR-T Characterization Panel (NanoString Technologies, Seattle, WA). Data were analyzed using the Rosalind platform (OnRamp Bio, San Diego, CA) for gene clustering, pathway enrichment, and differential gene expression. NanoString data have been deposited on the NCBI Gene Expression Omnibus (GEO) server under the accession number GSE206373.

### CAR T cell proliferation after repeated exposure to antigen

Luciferase-expressing target cells were added at 2 × 10^5^ per well and cocultured with 6 × 10^5^ CAR^+^ cells at a predetermined E:T ratio of 3:1 in 24-well cell culture plates in 1.5 ml of assay medium without IL-2. Each condition was performed in duplicates. Every 2 or 3 days, cells were thoroughly mixed by gentle pipetting, and 400 μl of cell suspension from each well was harvested and replaced with 2 × 10^5^ luciferase-expressing target cells in 400 μl of assay medium. Of the harvested cell suspension, 200 μl was discarded, and 200 μl was subjected to CAR T cell enumeration and phenotyping by flow cytometry.

### CAR T cell proliferation following target withdrawal

Effector cells and MM.1S target cells were cocultured at 3:1 E:T ratio for 48 hours. Cells were then thoroughly mixed by gentle pipetting, and a sample was taken for flow cytometry analysis. Fresh target cells were added to each well, and cells were cultured for 48 hours, and this step was repeated on days 4 and 7. On day 9, cells were transferred to a new plate and for each test sample, one well received MM.1S target cells (target exposure wells) or T cell assay medium alone (target withdrawal wells). Every 2 to 4 days, a small sample of cells was harvested for flow cytometry analysis and CAR T cell enumeration, and the remaining cells were either re-exposed to target by addition of fresh target cells (target exposure wells) or kept in the absence of target by addition of T cell assay medium alone (target withdrawal wells). This step was performed for several weeks. For comparison, BCMA CAR T cells were tested with or without addition of recombinant human IL-15 (10 ng/ml, twice weekly). Absolute counts of CAR^+^ cells were determined by flow cytometry analysis, and CAR T cell expansion was expressed as fold change relative to day 0.

### Cytokine release assay

A total of 2.5 × 10^5^ CAR^+^ cells or control nontransduced T cells were mixed with a same number of BCMA-overexpressing REH target cells (REH-BCMA) in a total of 500 μl of assay medium in 24-well cell culture plates. As a control, effector cells were mixed with a same number of BCMA-negative REH cells. Each condition was performed in duplicates. After overnight incubation at 37°C, plates were centrifuged at 400*g* for 5 min and 200 μl of supernatant was transferred to a 96-well U-bottom plate and stored at −80°C. After at least 24 hours at −80°C, the supernatants were thawed, and the concentrations of interferon-γ, IL-2, tumor necrosis factor–α, IL-2Rα, IL-6, and granulocyte-macrophage colony-stimulating factor were measured using the U-PLEX Biomarker Group 1 (hu) Assay Kit (Meso Scale Discovery, Rockville, Maryland), following the manufacturer’s protocol.

### Cytokine independent proliferation assay

Cryopreserved TurboCAR T cells were thawed and suspended in X-Vivo 15 medium supplemented with 10% FCS. Cells were counted and plated at 5 × 10^5^ cells/ml in triplicate wells of a 24-well tissue culture plate and cultured in a humidified incubator at 37°C and 5% CO_2_. As a control, BCMA CAR T cells were cultured with or without recombinant human IL-15 (10 ng/ml, once weekly). Once weekly, a small sample of cells was harvested for flow cytometry analysis and CAR T cell enumeration, and the remaining cells were fed with fresh medium. This step was performed for several months. Absolute counts of CAR^+^ T cells were determined by flow cytometry analysis, and CAR T cell expansion was expressed as a fold change in CAR^+^ T cell numbers relative to that at the start of the assay. Values were expressed as means ± SEM.

### In vivo models

All animal studies were performed in accordance with regulations and established guidelines and were reviewed and approved by an Institutional Animal Care and Use Committee. Eight- to 12-week-old female NOD.Cg-Prkdcscid Il2rgtm1Wjl/SzJ (NSG) mice were obtained from the Jackson Laboratory (Bar Harbor, ME). For the orthotopic multiple myeloma model, NSG mice were irradiated with 1 gray 1 day before 5 × 10^6^ luciferase-labeled MM.1S cells/mouse were injected intravenously. Twice weekly, tumor bioluminescence was determined using the IVIS Lumina imaging system (PerkinElmer, Boston, MA), and body weights were measured. Mice were randomized on the basis of tumor bioluminescence before CAR T cell infusion 15 days after tumor implantation. For subcutaneous tumor models, 3 × 10^6^ Molp-8 cells were implanted subcutaneously, and tumor growth was monitored twice weekly by caliper measurements using a digital caliper. Tumor size was calculated using the formula Tumor volume = (width^2^ × length/2). Mice were randomized into groups on the basis of tumor volume before CAR T cell infusion. For persistence studies in nontumor-bearing mice using CBR-labeled cells, mice received a single dose of 7 × 10^6^ CAR^+^ cells (BCMA CAR T and BCMA TurboCAR T cell groups) or 1.8 × 10^7^ CBR-only T cells (control group) via tail vein injection. For persistence studies in mice bearing subcutaneous tumors, mice received a mix of CBR-labeled and unlabeled CAR T cells (0.5 × 10^6^ CBR^+^CAR^+^ cells mixed with 9.5 × 10^6^ unlabeled CAR^+^ cells) via tail vein injection. On the day of CAR T cell dosing, cryopreserved CAR T cells and nontransduced T cells were thawed and counted according to standard procedures. Total T cell numbers were kept constant across all groups by normalizing with nontransduced T cells. Cells were resuspended in serum-free RPMI, and the indicated dose of CAR^+^ T cells were injected intravenously in a blinded fashion. Where indicated, animals received intraperitoneal injections of rituximab or vehicle control (10 mg/kg). To enumerate CAR T cells, blood was collected into EDTA-coated tubes using submandibular bleeds, and 50 μl was used for flow cytometry. Animals were euthanized when they exhibited disease model–specific end points such as hind leg paralysis, ruffled fur, tumor volumes approaching 2500 mm^3^, or body weight loss exceeding 20%.
