# Supplementary material for: Constitutive Turbodomains enhance expansion and antitumor activity of allogeneic BCMA CAR T cells in preclinical models
Source: Sci Adv. 2023 Aug 4;9(31):eadg8694. doi: 10.1126/sciadv.adg8694 (PMC10403208; doi:10.1126/sciadv.adg8694)
Supplement: Supplementary file 1 — Supplementary Materials and Methods Figs. S1 to S11 Table S1 [file sciadv.adg8694_sm.pdf]

Science Advances
AAAS

Supplementary Materials for

Constitutive Turbodomains enhance expansion and antitumor activity of allogeneic BCMA CAR T cells in preclinical models

Regina J. Lin *et al.*

Corresponding author: Regina J. Lin, regina.lin.junhui@gmail.com; Cesar Sommer, cesar.sommer@allogene.com

Sci. Adv. **9**, eadg8694 (2023)
DOI: 10.1126/sciadv.adg8694

This PDF file includes:

Supplementary Materials and Methods
Figs. S1 to S11
Table S1

SUPPLEMENTARY MATERIALS AND METHODS

Antibodies, flow cytometry analysis and FACS sorting

Antibodies used for T cell characterization were anti-hCD3-BUV395 (clone UCHT1, BD Biosciences, San Jose, CA), anti-hCD4-BV605 (clone OKT4, Biolegend), anti-hCD8-BV510 (clone RPA-T8, Biolegend), anti-hCD62L-PE/Cy7 (clone DREG-56, Biolegend), anti-hCD45RO-BV785 (clone UCHL1, Biolegend), anti-hPD1-APC (clone EH12.2H7, Biolegend), anti-hTim3-BV711 (clone F38-2E2, Biolegend), anti-hLag3-PerCP/Cy5.5 (clone 11C3C65, Biolegend), anti-hTCRαβ-APC (clone BW242/412, Miltenyi Biotec), anti-hCD52-FITC (clone HI186, Miltenyi Biotec), anti-hIFNγ-PE/Cy7 (clone 4S.B3, Biolegend, San Diego, CA), anti-hIL-2-PEDazzle594 (clone MQ1-17H12, Biolegend, San Diego, CA), anti-hTNFα-BV785 (clone Mab11, Biolegend, San Diego, CA), anti-Ki67-BV711 (Biolegend, San Diego, CA), and anti-h/mStat5 (pY694)-AlexaFluor647 (clone 47/Stat5(pY694), BD Biosciences, San Jose, CA). For detection of v5-tagged EGFRvIII CAR, either an anti-v5 tag-FITC (polyclonal, Novus Biologicals) or a soluble EGFRvIII-PE (produced in-house) was used. For detection of BCMA CARs, an anti-idiotype-PE (produced in-house) was used. Where applicable, mouse blood samples were treated twice using ACK lysing buffer (Thermo Fisher Scientific, Waltham, MA) for red blood cell removal. Dead cells were detected using the Zombie NIR Fixable Viability Kit (BioLegend, San Diego, CA). Cells were stained for surface markers using standard flow cytometry protocols in 1x PBS containing 2% FBS and 2 mM EDTA supplemented with human TruStain Fc blocking reagent (BioLegend, San Diego, CA). For intracellular cytokine detection, cells were fixed and permeabilized using the Cytofix/Cytoperm Fixation/Permeabilization Solution Kit (BD Biosciences, San Jose, CA) and stained according to manufacturer's instructions. For intracellular pSTAT5 detection, cells were serum starved for 4 hours at 37°C in RPMI containing GlutaMax and HEPES (Gibco, Thermo Fisher Scientific, Waltham, MA) prior to staining for cell surface markers. Cells were then fixed in 4% paraformaldehyde, washed and permeabilized with 100% methanol before intracellular staining for pSTAT5. To enumerate CAR T cells, samples were supplemented with counting beads (Thermo Fisher Scientific, Waltham, MA). All samples were acquired using Fortessa LSRII (BD Biosciences, San Jose, CA) or CytoFLEX (Beckman Coulter, Brea, CA) flow cytometers. For

FACS sorting, BCMA CAR T cells were stained using the Zombie NIR Fixable Viability Kit (BioLegend, San Diego, CA), followed by the anti-idiotype-PE. Viable CAR^+ cells were bulk sorted on the FACS Aria II Cell Sorter (BD Biosciences, San Jose, CA).

Lentivirus production, concentration, titration and quantification

To generate lentiviral supernatants, HEK293T cells were plated at 0.45 million cells per ml in 2 ml of DMEM (Gibco, Thermo Fisher Scientific, Waltham, MA) supplemented with 10% FBS (GE Healthcare, Pittsburgh, PA) per well of a 6-well plate on Day -1. On Day 0, the lentivirus was prepared by mixing lentiviral packaging vectors 1.5 µg psPAX2, 0.5 µg pMD2G, and 0.5 µg of the appropriate transfer CAR/TurboCAR vector in 250 µl Opti-MEM per well of the 6-well plate ("DNA mix"). 10 µl Lipofectamine 2000 in 250 µl Opti-MEM was incubated at room temperature for 5 minutes and then added to the DNA mix. The mixture was incubated at room temperature for 20 minutes and the total volume of 500 µl was slowly added to the sides of the wells containing HEK293T cells. On Day 1, Lipofectamine-containing supernatants were removed and replaced with X-Vivo15 medium (Lonza, Basel, Switzerland), supplemented with 10% FBS (GE Healthcare, Pittsburgh, PA). On Day 2, crude lentiviral supernatants were harvested, filtered through a 0.45 µm filter, and were either used fresh for human CAR T cell generation, or concentrated 25x using Lenti-X Concentrator (Takara Bio, Shiga, Japan) according to the manufacturer's instructions and stored at -80°C until use. Where applicable, lentiviral titration was performed on Jurkat cells. Briefly, on Day 0, 2×10^4 Jurkat cells were transduced with serially diluted lentivirus in 96-well U-bottom plates. On Day 2, the percentage of CAR^+ Jurkat cells was determined by flow cytometry. Titer was calculated using the volume of lentivirus yielding 10% CAR^+ cells.

Complement-dependent cytotoxicity (CDC) assay

$1x10^5$ cells were incubated in RPMI 1640 medium supplemented with 10% FCS for 1 h in the presence of 12.5% baby rabbit complement (Bio-Rad, Hercules, CA) and rituximab at 0, 10, 30, and 100 µg/ml. Cells were then collected by centrifugation, washed with FACS buffer, and stained for flow cytometry analysis. The percentages of depleted CAR^+ cells were normalized between 0 and 100% relative to wells without

antibody (set to 0% depletion). CDC was expressed as the increase in the % of depleted CAR^+ cells relative to the control using the formula 100 – [100 x (CAR^+ cells test /CAR^+ cells control)].

Antibody-dependent cellular cytotoxicity (ADCC) assay

Natural killer (NK) cells were isolated using StemCell Technologies NK Cell Enrichment Cocktail following the manufacturer's protocol. NK cells were then counted, suspended at 4×10^6 cells/ml in RPMI 1640 medium supplemented with 10% FCS, and activated using 1000 IU/ml IL-2 for 2 days in a humidified incubator at 37°C and 5% CO_2. On Day 2, NK cells were collected by centrifugation and suspended in the same medium. CAR T cells were labeled with CellTrace Violet dye following the manufacturer's protocol and co-cultured with NK cells at an E:T of 10 for one hour. Rituximab antibody was added at increasing concentrations ranging from 0.1 to 100 µg/ml. Cells were then collected by centrifugation, washed with FACS buffer, and stained for flow cytometry analysis. The percentages of depleted CAR^+ cells were normalized between 0 and 100% relative to wells with CAR T cells without antibody and NK cells (set to 0% depletion). ADCC was expressed as the increase in the % of depleted CAR^+ cells relative to the control using the formula 100 – [100 x (CAR^+ cells test /CAR^+ cells control)]. Two NK cell donors and CAR T cells produced from 3 donors were tested in this study.

SUPPLEMENTARY FIGURES

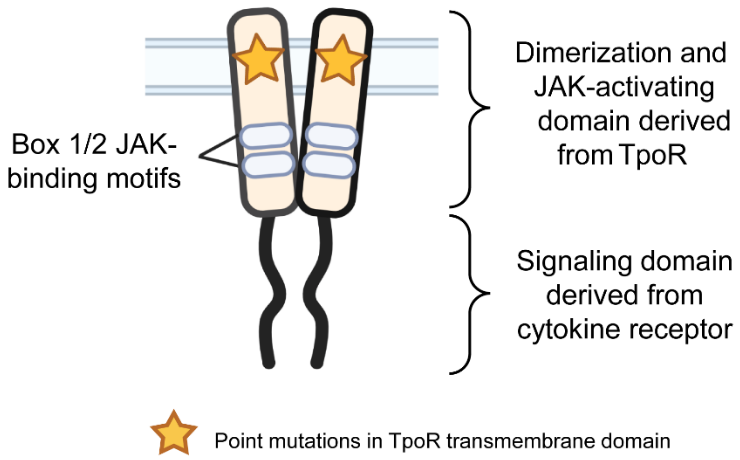

Fig. S1: Schematic of a Turbodomain. Turbodomains are membrane-bound chimeric cytokine receptors comprised of a dimerization and JAK-activating domain derived from TpoR, fused to intracellular signaling domain(s) derived from the cytokine receptor(s) of interest. Mutations in the transmembrane domain induce ligand-independent receptor dimerization and activation.

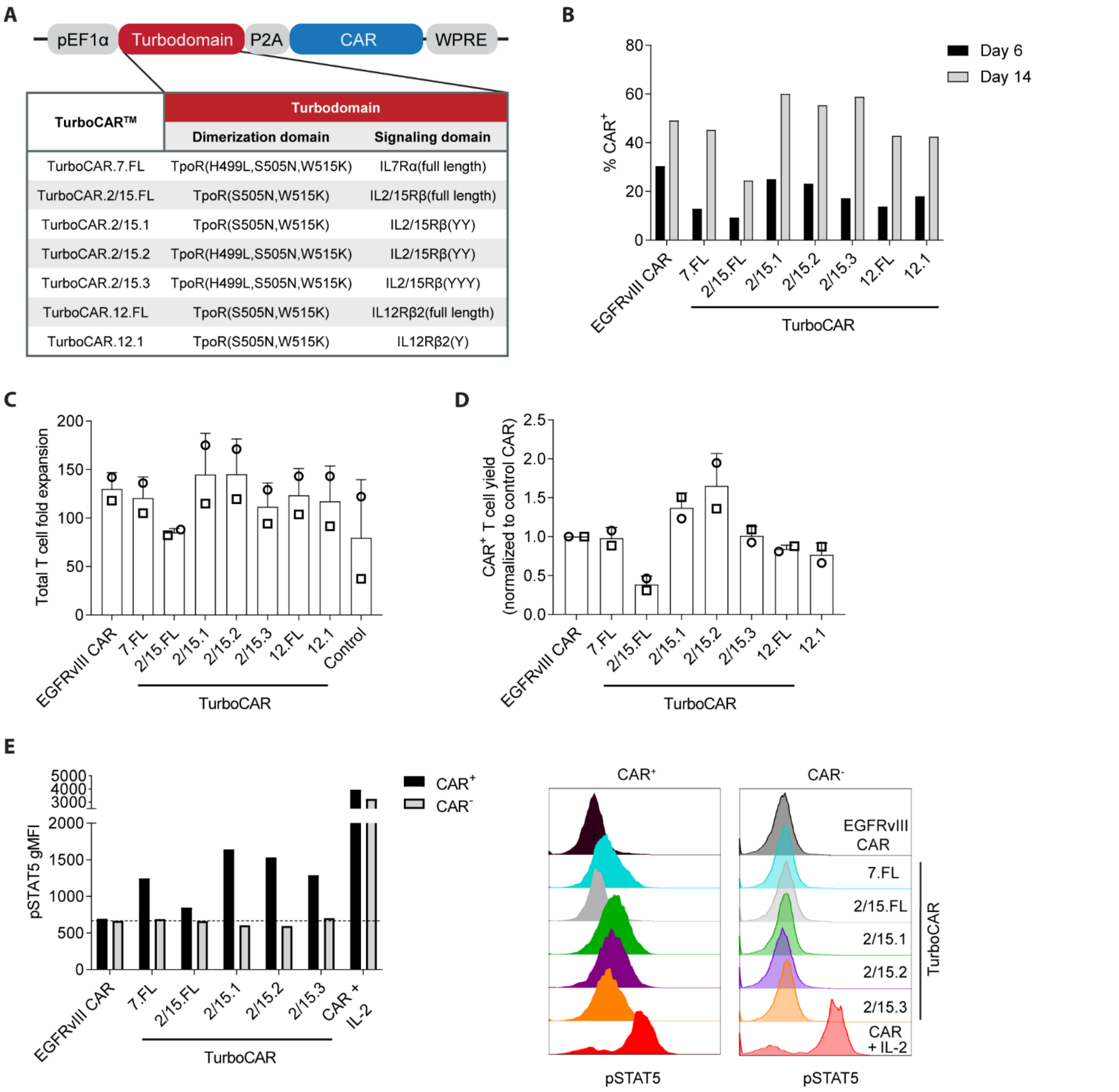

Fig. S2: Generation and phenotype of EGFRvIII TurboCAR T cells with different signaling domains. (A) Schematic of a TurboCAR lentiviral construct and list of selected TurboCAR constructs co-expressing a CAR and the indicated Turbodomain. **(B)** Initial transduction efficiency (Day 6) and CAR percentage at end-of-process (Day 14), **(C)** total T cell expansion, and **(D)** CAR$^+$ T cell yield are shown. **(E)** Turbodomain signaling activity was assessed in transduced T cells by intracellular flow cytometry staining for pSTAT5. EGFRvIII CAR$^-$ T cells were used as background gating controls and pSTAT5 gMFI

equal or below that of EGFRvIII CAR^{-} T cells (dotted line) was considered background. Data are shown for one representative of two donors (B and E) or as mean ± SD of two donors with open circles and squares representing different donors (C and D).

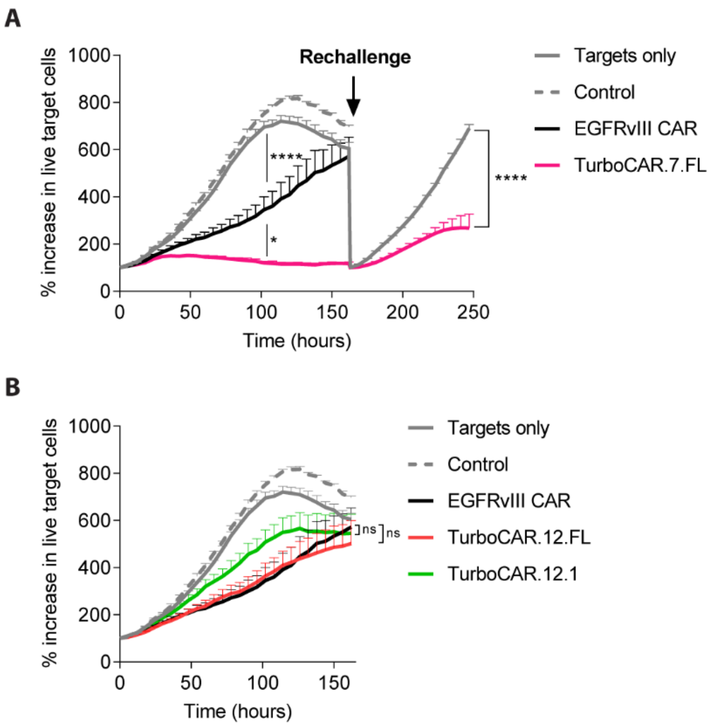

Fig. S3: The EGFRvIII TurboCAR.7.FL but not the EGFRvIII TurboCAR.12 variants enhanced cytotoxicity in vitro. Cytotoxicity of TurboCAR.7.FL **(A)** and TurboCAR.12 **(B)** variants against nuclear GFP-labeled U87KO-EGFRvIII target cells was assessed in an Incucyte assay at an effector to target ratio (E:T) of 1:3. Where indicated, CAR T cells were rechallenged with fresh target cells. The percentage increase in live target cells was determined by normalizing target cell counts at each timepoint to that at time=0. Data are mean ± SEM of triplicate wells from one representative of two donors. Statistical significance determined by ordinary one-way ANOVA with Dunnett's test. *$P < 0.05$, ****$P < 0.0001$, ns: not significant.

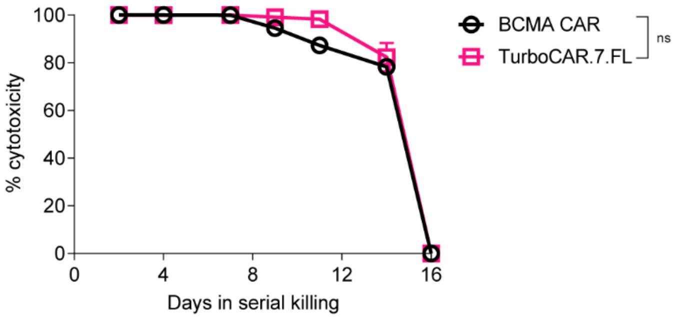

Fig. S4: Long-term cytotoxicity of BCMA TurboCAR.7.FL variant. Effector cells were co-cultured with MM.1S-Luc-GFP target cells at an E:T of 10:1 and fresh target cells were added every 2-3 days. Target cell killing was determined by luminescence readout. Data are presented as mean ± SEM of triplicates from one of two donors. Statistical significance determined by unpaired t-test of area under the curve (AUC). ns: not significant.

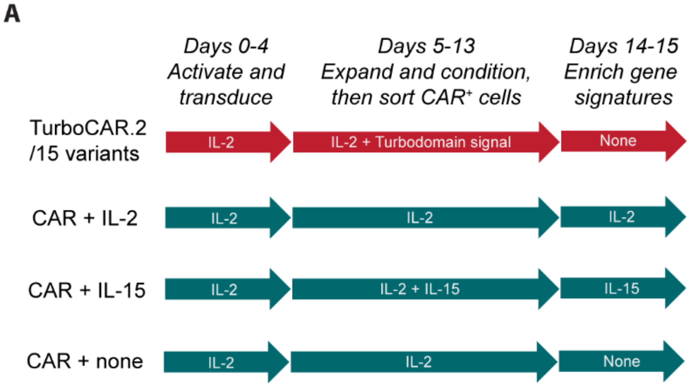

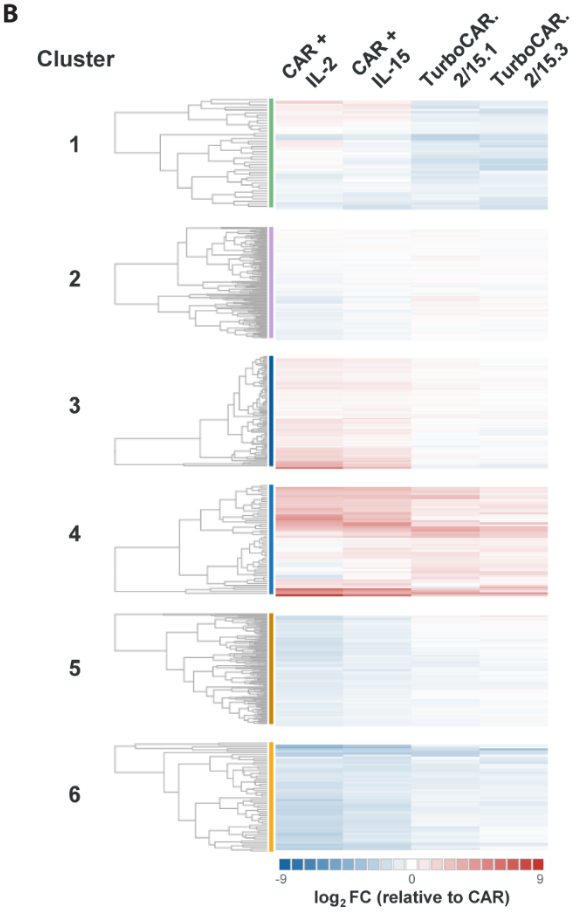

C

Cluster	MSigDB Pathway/Hallmark	p-Adj
1	Interferon gamma response	0.37892
2	Oxidative phosphorylation	0.42341
3	mTORC1 signaling	0.00118
4	IL-2 STAT5 signaling	0.06123
5	Allograft rejection	0.59169
6	TNFA signaling via NKFB	0.25189

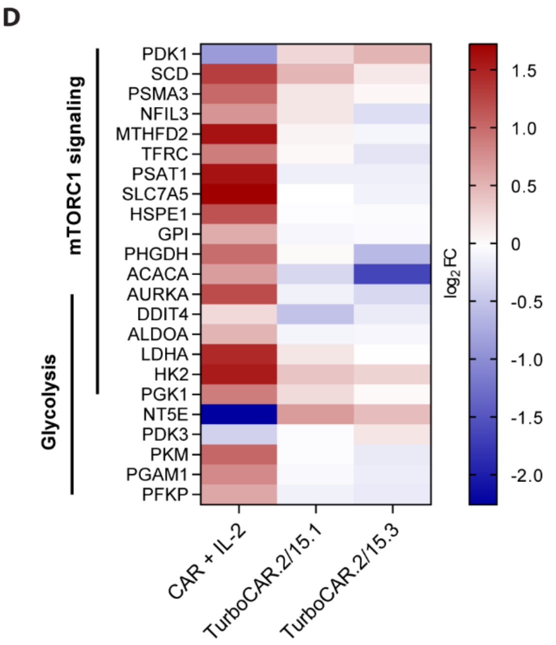

Fig. S5: Meta-analysis identifies gene clusters and pathways differentially represented as a result of cytokine treatment or Turbodomain signaling. (A) Experimental design for gene expression analysis using the Nanostring nCounter Human CAR T Characterization panel. TurboCAR.2/15 T cells were generated by expansion in IL-2 as per standard protocol. To mimic the aggregate of cytokine signaling received by TurboCAR.2/15 T cells, unmodified CAR T cells were expanded in either IL-2 alone

(100 U/ml), or a combination of IL-2 (100 U/ml) and IL-15 (10 ng/ml). At the end of expansion, CAR^+ T cells were FACS-sorted and cultured for an additional 2 days to enrich for Turbodomain- or cytokine-induced gene signatures before Nanostring gene expression profiling and analysis. **(B)** Gene expression was normalized to that of control CAR T cells cultured without cytokines from days 14-15 and data from two donors were pooled for meta-analysis using the Rosalind platform. Heat map shows log_2-fold changes in gene expression relative to CAR T cells. **(C)** Geneset enrichment analysis from the Molecular Signature Database (MSigDB) Hallmark collection. The pathway most closely associated to each cluster based on lowest p-Adj and size of enriched gene set is shown. **(D)** Geneset enrichment analysis using the MSigDB Hallmark database reveal that the differentially expressed genes were associated with mTORC1 signaling, glycolysis, or both pathways.

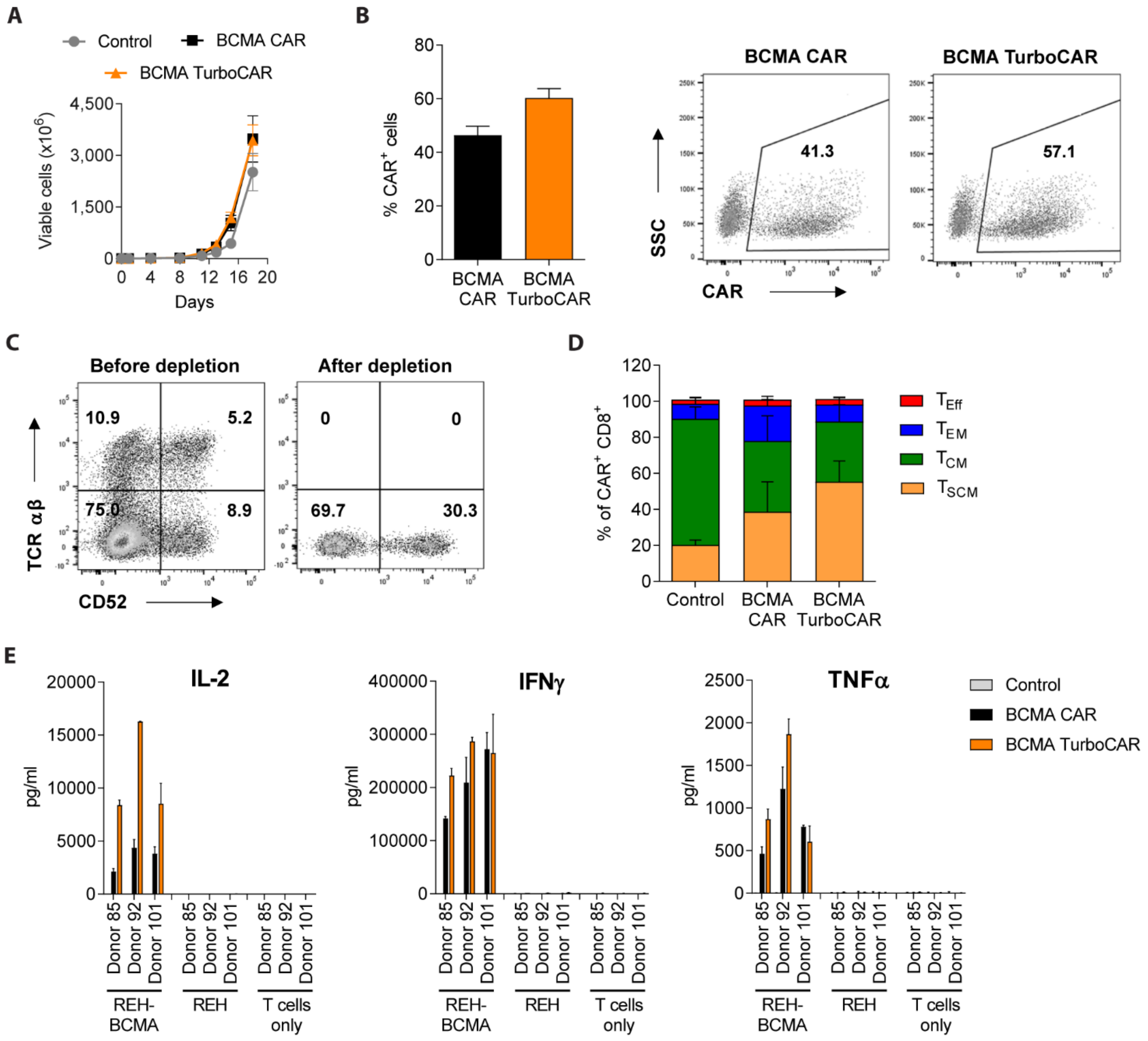

Fig. S6: Efficient production of allogeneic BCMA TurboCAR T cells using healthy-donor PBMCs. **(A)** Cell expansion and viability were measured at the indicated timepoints during CAR T cell production. Data are shown as mean ± SEM of triplicate wells from one representative of three donors. **(B)** Following activation, T cells were transduced with a lentiviral vector for expression of the BCMA CAR and Turbodomain followed by knockout of the TRAC and CD52 genes and expansion in G-Rex vessels. Bar graphs show the percentage of T cells among live cells expressing the BCMA CAR at end of process (mean plus SEM of 3 donors). FACS plots for one representative donor are shown. **(C)** Gene editing efficiency was assessed by flow cytometry analysis of TCRαβ and CD52 expression on TurboCAR T

cells before and after MACS-based depletion of residual TCRαβ$^+$ cells (representative data from one donor shown). **(D)** At the time of cryopreservation, BCMA TurboCAR T cells contained a high frequency of cells expressing memory markers, indicative of high proliferative potential. Bars represent mean plus SD (n=3 donors). **(E)** The concentrations of IFNγ, IL-2 and TNFα were measured by MSD following co-culture of effector cells alone, with BCMA-negative REH cells, or with BCMA-overexpressing REH cells (REH-BCMA) at 1:1 ratio for 24 hours. Data are shown as mean plus SD of duplicate wells for each donor.

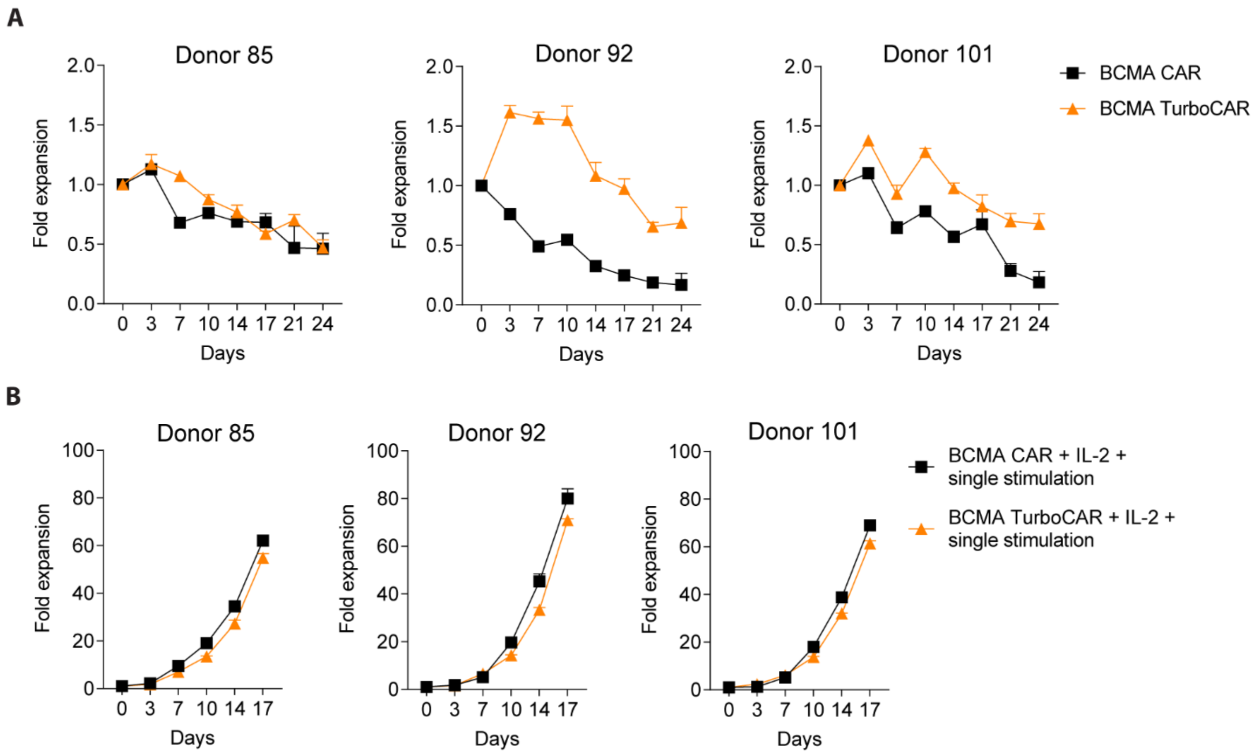

Fig. S7: Allogeneic BCMA TurboCAR T cells do not display IL-2 independent growth. (A) The proliferative capacity of BCMA TurboCAR T cells cultured in the absence of IL-2 or target cells was assessed by counting total viable cells over time. Fold expansion at each time point was calculated relative to day 0. Graphs show fold expansion of cells as measured in quadruplicate wells (mean ± SEM) for each CAR T cell product. **(B)** Graphs show a similar analysis of CAR T cell expansion following a single exposure to target cells in the presence of IL-2 (50 IU/ml, added twice a week).

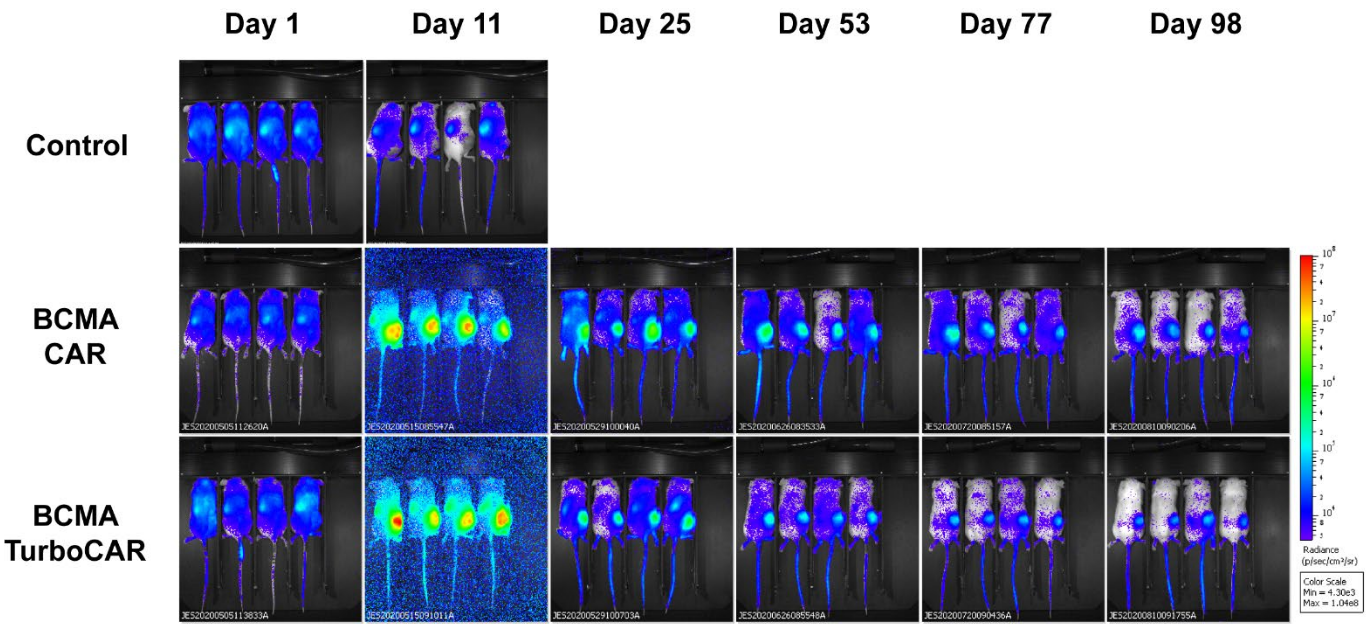

Fig. S8: Allogeneic BCMA TurboCAR T cells show progressive localization to the site of tumor implantation. Molp-8 tumor cells were implanted subcutaneously on the right flank of NSG mice ($3x10^6$ cells/mouse) and tumor volume was measured using calipers. When tumors reached approximately 180 mm^3, a mix of CBR-labeled and unlabeled TurboCAR T cells ($0.5x10^6$ CBR^+CAR^+ cells and $9.5x10^6$ unlabeled CAR^+ cells) was injected intravenously into mice and bioluminescence was measured for several weeks. Images were taken at the indicated timepoints and are representative of the group.

A

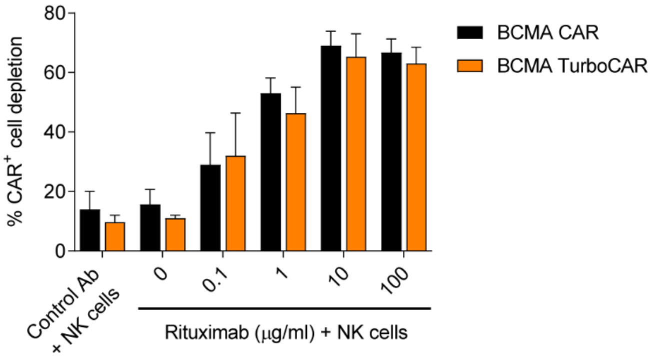

B

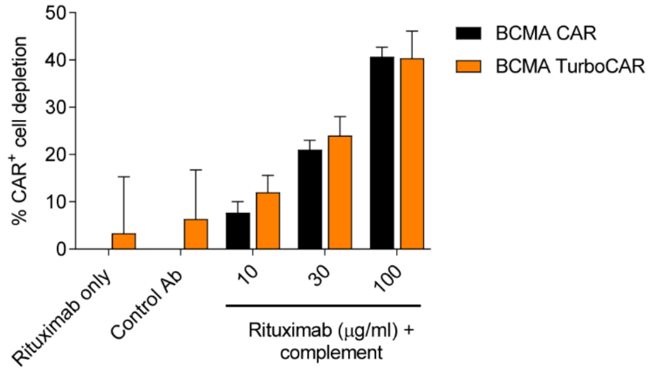

Fig. S9: Allogeneic BCMA TurboCAR T cells can be depleted by rituximab via ADCC and CDC. (A) BCMA TurboCAR T cells were incubated with activated NK cells and various concentrations of the anti-CD20 antibody rituximab or an isotype control antibody (100 µg/ml). After one hour, cells were stained for CAR expression and analyzed by flow cytometry to determine the percentages of depleted CAR^+ cells relative to wells containing CAR T cells cultured without antibody and NK cells. The graph shows the percentage of depleted CAR^+ cells (mean ± SEM; n=3 donors). Statistical analysis using-2-way ANOVA and Sidak's multiple comparison revealed no significant difference between BCMA TurboCAR T cells and BCMA CAR T cells. NK cells from two donors were tested in the assay, and data from one representative donor are shown. **(B)** BCMA TurboCAR T cells were incubated with baby rabbit complement and various concentrations of rituximab or an isotype control antibody (100 µg/ml) for one hour. Cells were washed and stained for CAR expression for flow cytometry analysis. The graph shows the percentage of depleted CAR^+ cells (mean ± SEM; n=3 donors). No significant differences in CDC were observed between BCMA

TurboCAR T cells and BCMA CAR T cells as determined by 2-way ANOVA test with Sidak's multiple comparison analysis.

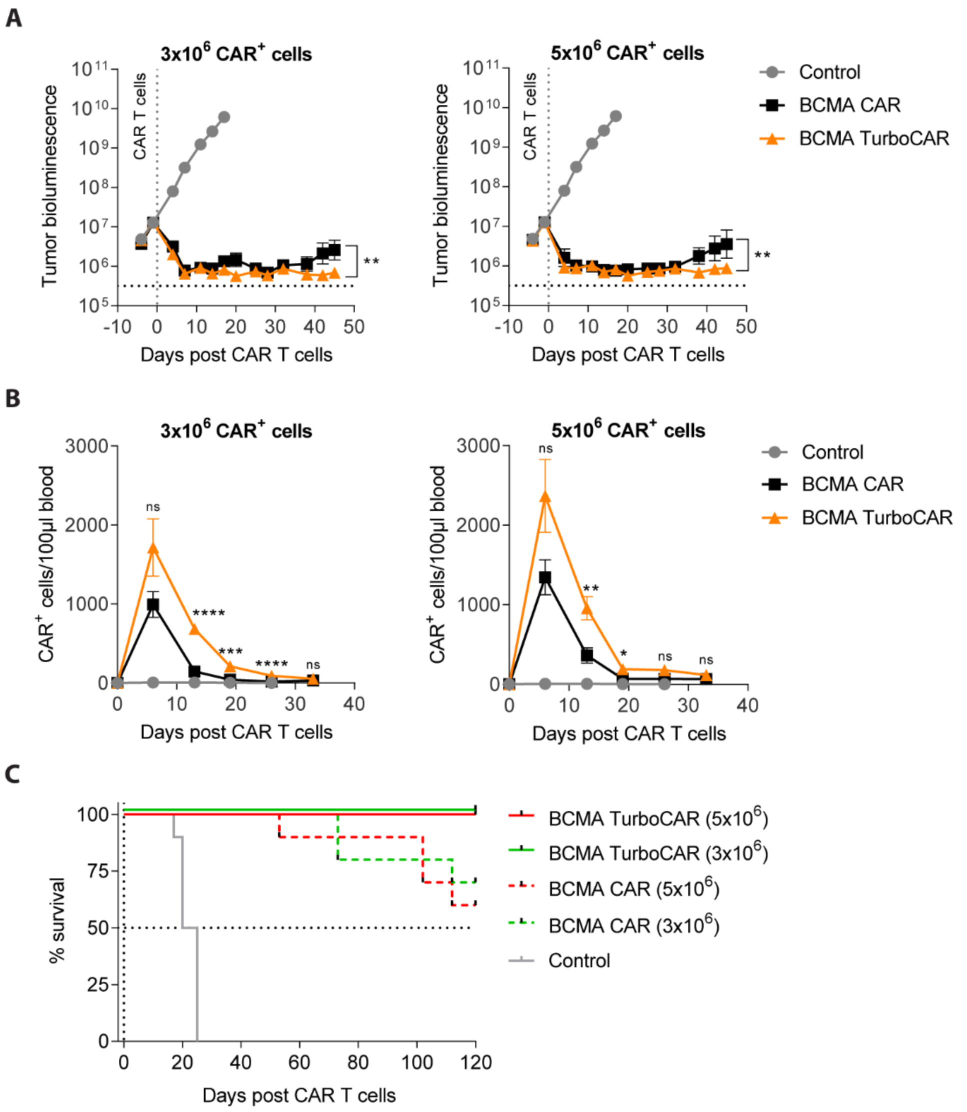

Fig. S10: Allogeneic BCMA TurboCAR T cells exhibit enhanced activity and extend survival in a mouse model of multiple myeloma. (A) NSG mice received $5x10^6$ luciferase-labeled MM.1S cells via tail vein injection and tumor growth was monitored by bioluminescence. Animals were randomly assigned into treatment groups 15 days after tumor implantation and treated with either $3x10^6$ or $5x10^6$ CAR^+ cells. Data are mean ± SEM (n=10)**.** A two-tailed, paired t test for all timepoints was used for comparison of the different treatments. Data are mean +/- SEM. **(B)** CAR^+ cell expansion post infusion was evaluated by flow cytometry analysis of mouse peripheral blood at the indicated timepoints. A Student's t-test for each timepoint, without multiple comparison correction, was used for comparison. Data are mean ± SEM. *P <

0.05, $^{**}P < 0.01$, $^{***}P < 0.001$, $^{****}P < 0.0001$, ns: not significant **(C)** Overall survival of mice in each group.

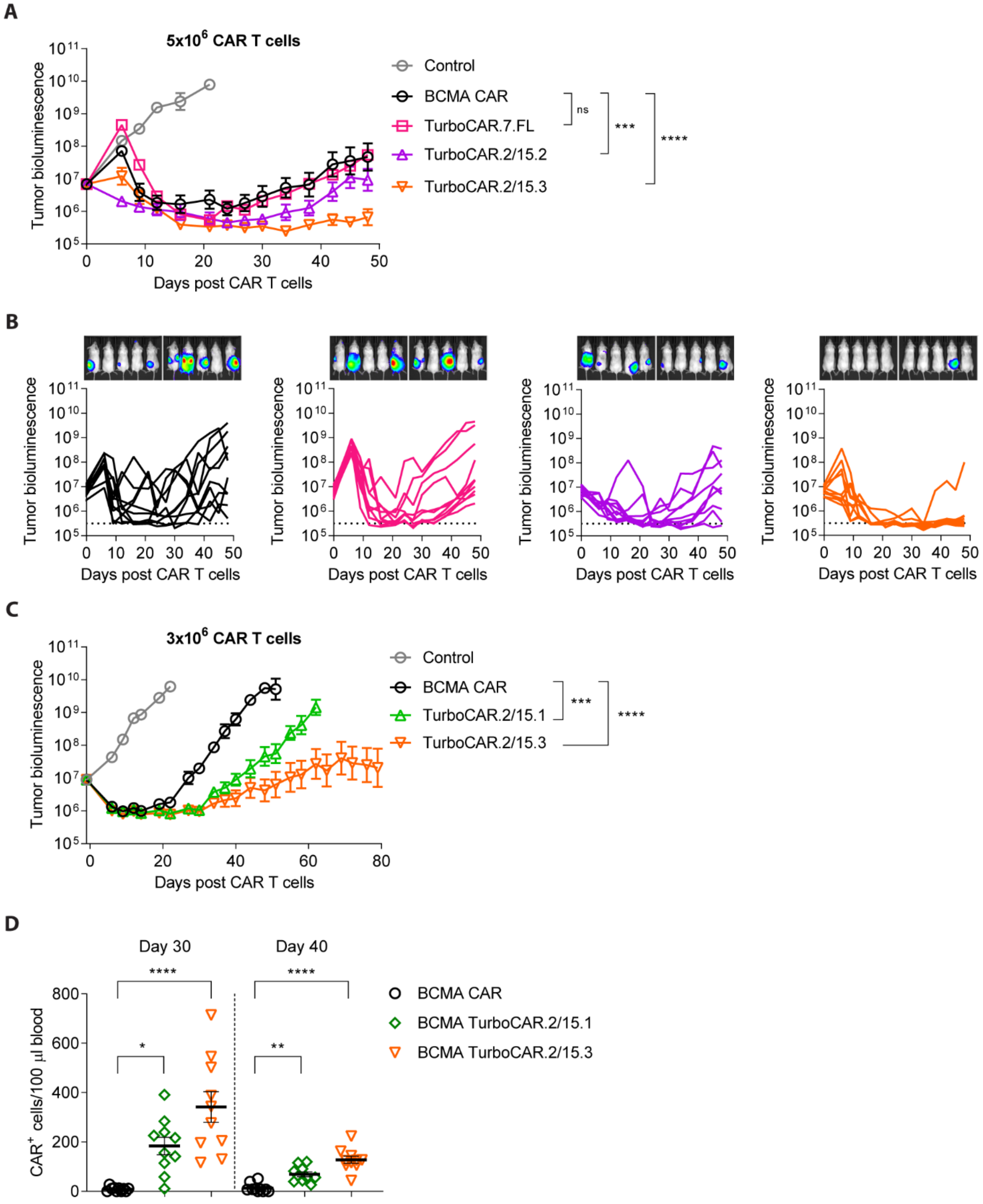

Fig. S11: Allogeneic BCMA TurboCAR.2/15 T cells show enhanced potency, persistence and anti-tumor activity. (A) NSG mice received 5x10^6 luciferase-labeled MM.1S cells via tail vein injection and tumor growth was monitored by bioluminescence. BCMA TurboCAR T cells (5x10^6 CAR$^+$ cells) were infused intravenously 15 days later. Data are mean ± SEM (n=10). **(B)** Tumor bioluminescence of

individual mice and images showing extramedullary relapse on Day 48. **(C)** NSG mice received 5x10^6 luciferase-labeled MM.1S cells via tail vein injection and tumor growth was monitored by bioluminescence. BCMA TurboCAR T cells (3x10^6 CAR^+ cells) were infused intravenously 15 days later. Data are mean ± SEM (n=10). **(D)** On the indicated days, CAR^+ cells in peripheral blood were enumerated using flow cytometry. Data are mean ± SEM. Statistical significance determined by RM one-way ANOVA with Dunnett's test from (A) Days 6-48 and (C) 5-51, and by (D) one-way ANOVA with Dunnett's test on Day 30 or 40 (*P = 0.05-0.01, **P = 0.01-0.001, ***P = 0.001-0.0001, ****P < 0.0001, ns = not significant).

SUPPLEMENTARY TABLES

Table S1: Turbodomain sequences

Turbodomain	Amino acid sequence
**TurboCAR.7.FL**	SDPTRVETATETAWISLVTALLLVLGLNAVLGLLLLRKQFPAHYRRLRHALW PSLPDLHRVLGQYLRDTAALSPPKATVSDTCEEVEPSLLEILPKSSERTPLPL ARDEVEGFLQDTFPQQLEESEKQRLGGDVQSPNCPSEDVVITPESFGRDS SLTCLAGNVSACDAPILSSSRSLDCRESGKNGPHVYQDLLLSLGTTNSTLPP PFSLQSGILTLNPVAQGQPILTSLGSNQEEAYVTMS SFYQNQ
**TurboCAR.2/15.FL**	SDPTRVETATETAWISLVTALHLVLGLNAVLGLLLLRKQFPAHYRRLRHALW PSLPDLHRVLGQYLRDTAALSPPKATVSDTCEEVEPSLLEILPKSSERTPLPL VTQLLLQQDKVPEPASLSSNHSLTSCFTNQGYFFFHLPDALEIEACQVYFTY DPYSEEDPDEGVAGAPTGSSPQPLQPLSGEDDAYCTFPSRDDLLLFSPSLL GGPSPPSTAPGGSGAGEERMPPSLQERVPRDWDPQPLGPPTPGVPDLVD FQPPPELVLREAGEEVPDAGPREGVSFPWSRPPGQGEFRALNARLPLNTD AYL SLQELQGQDPTHLV
**TurboCAR.2/15.1**	SDPTRVETATETAWISLVTALHLVLGLNAVLGLLLLRKQFPAHYRRLRHALW PSLPDLHRVLGQYLRDTAALSPPKATVSDTCEEVEPSLLEILPKSSERTPLPL DEGVAGAPTGSSPQPLQPLSGEDDAYCTFPSRDDLLLFSPSGQGEFRALN ARLPLNTDAYLSLQELQGQDPTHLV
**TurboCAR.2/15.2**	SDPTRVETATETAWISLVTALLLVLGLNAVLGLLLLRKQFPAHYRRLRHALW PSLPDLHRVLGQYLRDTAALSPPKATVSDTCEEVEPSLLEILPKSSERTPLPL DEGVAGAPTGSSPQPLQPLSGEDDAYCTFPSRDDLLLFSPSGQGEFRALN ARLPLNTDAYLSLQELQGQDPTHLV
**TurboCAR.2/15.3**	SDPTRVETATETAWISLVTALLLVLGLNAVLGLLLLRKQFPAHYRRLRHALW PSLPDLHRVLGQYLRDTAALSPPKATVSDTCEEVEPSLLEILPKSSERTPLPL QQDKVPEPASLSSNHSLTSCFTNQGYFFFHLPDALEIEACQDEGVAGAPTG SSPQPLQPLSGEDDAYCTFPSRDDLLLFSPSGQGEFRALNARLPLNTDAYL SLQELQGQDPTHLV
**TurboCAR.12.FL**	SDPTRVETATETAWISLVTALHLVLGLNAVLGLLLLRKQFPAHYRRLRHALW PSLPDLHRVLGQYLRDTAALSPPKATVSDTCEEVEPSLLEILPKSSERTPLPL VTPVFRHPPCSNWPQREKGIQGHQASEKDMMHSASSPPPPRALQAESRQ LVDLYKVLESRGSDPKPENPACPWTVLPAGDLPTHDGYLPSNIDDLPSHEA PLADSLEELEPQHISLSVFPSSSLHPLTFSCGDKLTLDQLKMRCDSLML
**TurboCAR.12.1**	SDPTRVETATETAWISLVTALHLVLGLNAVLGLLLLRKQFPAHYRRLRHALW PSLPDLHRVLGQYLRDTAALSPPKATVSDTCEEVEPSLLEILPKSSERTPLPL SDPKPENPACPWTVLPAGDLPTHDGYLPSNIDDLPSHEAPLADSLEELEPQ
